# Influence of effective thickness in elastic anisotropy and surface acoustic wave propagation in CoFeB/Au multilayer

**DOI:** 10.1038/s41598-025-08560-8

**Published:** 2025-07-15

**Authors:** A. V. Achuthan, S. Janardhanan, P. Kuświk, A. Trzaskowska

**Affiliations:** 1 Faculty of Physics and Astronomy, ISQI, Adam Mickiewicz University, Uniwersytetu Poznańskiego 2, 61-614 Poznan, Poland; 2 Institute of Molecular Physics, Polish Academy of Science, Smoluchowskiego 17, 60-179 Poznan, Poland

**Keywords:** Magnetic multilayers, Surface phonons, Brillouin scattering spectroscopy, Finite element method, Magnetic properties and materials, Surfaces, interfaces and thin films, Magnetic properties and materials

## Abstract

Surface acoustic waves (SAWs) in multilayered nanostructures represent a critical frontier in understanding material behavior at the nanoscale, with profound implications for emerging acoustic and spintronic technologies. In this study, we investigate the influence of the magnetic layer thickness on the propagation of surface acoustic waves in CoFeB-based multilayers. Two approaches to effective medium modelling are considered: treating the entire multilayer as a homogeneous medium and focusing on the region affected by light penetration. The elastic properties of the system are analyzed using Brillouin light scattering and numerical modelling, with a particular emphasis on the anisotropy of Young’s modulus and its dependence on CoFeB thickness. The results reveal a significant variation in surface acoustic wave velocity and elastic anisotropy as a function of the multilayer configuration, highlighting the role of the penetration depth in effective medium approximations. The best agreement with BLS measurements was obtained for the effective medium model limited to the optical penetration depth, highlighting the importance of light-matter interaction volume in SAW-based diagnostics. These findings provide valuable insights into the tunability of acoustic and spin-wave frequencies through structural modifications, which is crucial for developing high-performance resonators, surface acoustic wave filters, and spin-wave-based information processing devices.

**Supplementary Information:**

The online version contains supplementary material available at 10.1038/s41598-025-08560-8.

## Introduction

Surface acoustic waves (SAW) have intrigued researchers for centuries, with significant contributions to the field dating back to 1887, when Lord Rayleigh initiated studies on this phenomenon. SAWs are elastic waves that propagate along the surface of a material, with their intensity exponentially decaying with depth. There are different types of surface acoustic waves, including Rayleigh, Sezawa, Lamb, and Love waves. A well-known mode is the Rayleigh surface acoustic wave (R-SAW), which can propagate along the free surface of a semi-infinite solid. In homogeneous materials, R-SAWs are the dominant wave mode. However, in nonhomogeneous materials, such as a substrate with a layer on it, various types of surface waves can propagate^1^. Rayleigh surface waves exhibit distinct behaviors in isotropic versus anisotropic materials. In isotropic materials, R-SAWs are dispersive^2^while in anisotropic materials, the amplitude decays with depth in an oscillatory manner. Importantly, the properties of Rayleigh waves, including phase velocity, are highly dependent on the direction of propagation relative to the crystallographic orientation of the studied material. Additionally, the presence of layers can significantly alter the propagation characteristics of R-SAW, adding complexity to their behavior in anisotropic materials^1,3^. The dispersion relations of SAWs can be substantially modified through various means, including phononic crystals, spatial constraints, or external stress fields^4^.

Determining the velocity, dispersion, and anisotropy of SAWs offers a non-invasive, highly sensitive method for accurately measuring elastic parameters, allowing researchers to determine nonlinear elastic properties at interfaces between rough surfaces and assess the elastic characteristics of thin films. Moreover, studies on SAWs in magnetic thin films have provided valuable insights into magnon-phonon interactions, an essential element for advancing multifunctional spintronic devices. The implications of SAW research are extensive, with significant potential to drive innovation in materials science, spintronics, and nanotechnology, emphasizing the importance of ongoing research in this area^5,6^. Numerous experimental investigations have demonstrated that SAWs are widely applicable across various material systems. The unique characteristics of SAWs, such as high resistivity^7^ low power consumption^8^ and compatibility with integrated circuit technology^9^ have facilitated their adoption in a broad range of applications. Notable examples include surface flaw detection^10^ ultrasonic signal processing devices, Fourier-transform processors^11^ SAW sensors, especially for biological and chemical/microfluidic-based sensing applications^12,13^ structural health monitoring, telecommunications, damage detection in metallic structures^14^wireless passive thermometer^15^ communication applications^16^ and so on.

In the current technology for microsystem fabrication, silicon is the most employed material, on which various layers with distinct properties are deposited. From an application standpoint, a thorough understanding of the fundamental properties of such systems is therefore crucial. One such property is the elastic characteristics, which can be indirectly determined through investigations into SAW propagation. The importance of surface waves in modern electronics, spintronics, and phonon engineering highlights the role of multilayer systems, particularly magnetic structures, in SAW propagation and spin wave phenomena. Understanding the fundamental properties of multilayer structures, including elastic characteristics, is essential for the continued advancement of technology.

One of the methods used to determine SAW properties, such as dispersion and velocity anisotropy, is Brillouin light scattering (BLS)^17^. Due to its high-frequency resolution, flexibility with samples, and localized spatial capabilities, BLS emerges as a powerful technique for measuring material properties^18^. Notably, BLS is the only technique capable of studying SAW dynamics, as well as the propagation of longitudinal and transverse bulk waves and pseudo-surface waves in the GHz frequency range^5^. It is a well-established technique frequently employed in nondestructive testing to assess the elastic properties of bulk materials and thin films^19^.

In this study, we investigated thermally excited surface acoustic waves in Si/Ti/Au/CoFeB/Au heterostructures using BLS techniques, which are of particular interest for applications in memory media and magnetoresistive sensors. Previous studies have demonstrated that CoFeB/Au layers exhibit Dzyaloshinskii-Moriya interaction (DMI), and their magnetic properties can be significantly modified by adjusting the thickness of the layers^5^. This leads to a pertinent question regarding the behavior of surface acoustic waves in response to variations in the thickness of the CoFeB layer. This study employs BLS techniques to investigate thermally excited surface acoustic waves in Si/Ti/Au/CoFeB/Au heterostructures, which hold promise for applications in memory media and magnetoresistive sensors. Given the impact of the DMI on the magnetic properties of CoFeB/Au layers and their sensitivity to layer thickness, we explore how variations in CoFeB thickness affect surface acoustic wave behavior.

Despite the intriguing magnetic properties of CoFeB layers, the phononic characteristics of these multilayer systems have received insufficient attention. To address this gap, we employed high-resolution Brillouin light scattering spectroscopy to investigate the propagation of Rayleigh and Sezawa waves within these heterostructures. Our experimental findings were validated and complemented by numerical simulations using COMSOL Multiphysics software, which enabled us to estimate key elastic parameters such as Young’s modulus and Zener anisotropy parameters. This integrated approach provides a deeper understanding of the relationships between magnetic and acoustic properties in these complex heterostructures.

## Materials and methods

### The crystal

The Ti(4 nm)/Au(60 nm)/CoFeB(*t*_CoFeB_)/Au(2 nm) sample with different thicknesses of CoFeB (*t*_CoFeB_ = 0.8–2 nm) was deposited onto naturally oxidized Si (001) substrate using magnetron sputtering in Ar atmosphere at P_Ar_ = 1.4 × 10^−3^ mbar. The deposition was performed with base pressure < 2 × 10^−8^ mbar. The dimensions of the sample were 5 × 10 mm^2^. The CoFeB layer was sputtered from a Co_20_Fe_60_B_20_ target, the composition of which was earlier verified by energy-dispersive X-ray spectroscopy. The thicknesses of the Ti, Au, and CoFeB layers were controlled by selecting the appropriate deposition time, based on the deposition rate obtained from profilometer measurements for the calibration sample. The thickness of the individual components forming the multilayer material has been placed in parentheses next to each material. An amorphous phase of CoFeB was verified by an X-ray diffractometer in grazing incident configuration^5,20^.

### Experimental setup

Using the BLS technique, we can measure inelastic scattering between incident photons and thermal phonons (acoustic waves). Phonon characteristics are determined by measuring the incident light’s wavevector projection (*q*) and scattered light’s frequency shift (Δ*f*). Momentum conservation dictates that the acoustic phonon’s wavevector is equivalent to the in-plane projection of the incident light’s wavevector.

In our study, surface acoustic phonon propagation was investigated using a six-pass tandem Brillouin spectrometer (JRS Scientific Instruments), which provides a contrast of 10^1521^. The source of light was a 3.5 W laser, Coherent V6, emitting the second harmonics of light of the length *λ*_0_ = 532 nm. Brillouin Light Scattering experiments measure the relative frequency shifts, represented by Stokes and anti-Stokes components, that occur when laser light undergoes inelastic scattering by acoustic phonons. A detailed description of the experimental setup is found in Refs.^20,21^. The measurements were conducted in the backscattering geometry with *pp* polarization for both incident and scattered light. Both polarizations were confined to the sagittal plane of the sample, defined by the wave vector of the phonon and the normal to the sample surface.

The wavevector *q* is given by the following equation:

1 $$q = \frac{{4\pi \sin \theta }}{{\lambda _{0} }}$$

where *θ* is the angle between the incident light and the normal of the sample, *λ*_*0*_ is the wavelength of the laser light. As *θ* varies from 5° to 85° value of wavevector varies from 2 to 22.7 μm^−1^. The uncertainties are 0.04 GHz for the frequency and 0.001 μm^−1^ for the wavevector.

The correlation between *θ* angle and frequency of the SAW gives us the phase velocity υ, which can be found in the equation:

2 $$\upsilon = \frac{{\Delta f\lambda _{0} }}{{2\sin \theta }}$$

Brillouin spectroscopy’s high sensitivity enables detailed characterization of the dispersion relation governing SAW propagation in the samples under study.

## Finite-element method simulations

To investigate surface acoustic waves, we employed COMSOL Multiphysics software^22^which utilizes the finite-element method (FEM) to tackle complex coupled systems of partial differential equations. We used a frequency-domain study to reflect the experimental excitation of the system. In the materials analyzed, the intrinsic inhomogeneity, variable density, and specific attributes of the elastic tensor play a crucial role in determining the localization of surface modes and, consequently, the dispersion relations of the systems.

For a precise assessment of mode localization and dispersion characteristics, we modelled the silicon substrate as a uniform semi-infinite medium (z ≤ 0), with nanostructures positioned on its surface. To maintain consistency in our analysis, we assumed the substrate to be perfectly flat during the simulations.

The elastic tensor and mass density for the CoFeB were calculated using the experimental values of the elastic tensor for each component (cobalt^23–25^ iron^26,27^ and boron^28,29^) at room temperature with respect to the chemical concentration.

The calculations were performed for the elastic constants of all components which create the sample - look at Table 1.

**Table 1:** Elastic properties *c*_*ij*_ (GPa) and density *ρ* (kg/m^3^) of materials used in FEM simulations.

	Silicon^4^	Titanium^30^	Gold^4^	CoFeB^30^
*c*_11_	165.7	178	190	267
*c*_12_	63.9	80.9	161	85
*c*_44_	79.9	43.4	42.3	120
*ρ*	2331	4503	19,300	7000

The geometric parameters used in the simulations were aligned with the actual characteristics of the samples studied experimentally. Bloch–Floquet periodic boundary conditions (PBC) were applied along the *x*- and *y*-axes of the unit cell^4^. These PBCs were implemented on both faces of the unit cell to ensure consistent values of the elastic tensor components and density throughout the entire modelled structure. In the simulations, the height of the unit cell was linked to the wavelength of the acoustic wave travelling through the sample, specifically set at 20 times the wavelength of the SAW. The bottom surface of the unit cell was fixed to simulate the elliptical decay of the surface wave along the height of the sample. To determine the localization of surface modes, the intensity of individual modes was utilized as a parameter, following the relationship. In simulations of multilayer structures, effective medium models, and light penetration depth, a similar mesh density was applied to ensure consistency and comparability of the results.

To calculate the SAW intensity $\:I\left({f}_{i},{q}_{i}\right),$ measured in the experiment, we use the integral of the *z*-component of the displacement vector $\:{u}_{z}^{{f}_{i},{q}_{i}}$ for the selected mode *i*, at the selected frequency $\:{f}_{i}$ and wavevector $\:{q}_{i}$ investigated over the free surface *A* of the studied sample^31^:

3 $$I\left( {f_{i} ,q_{i} } \right) = \left| {\mathop \int \limits_{A} u_{z}^{{f_{i} ,q_{i} }} dA} \right|^{2}$$

where $\:{u}_{z}$ represents the *z*-component of the total displacement *u*, defined $\:u=\sqrt{{u}_{x}^{2}+{u}_{y}^{2}+{u}_{z}^{2}}$ and the integration is performed over the free surface of the system.

## Results

**Fig. 1:**
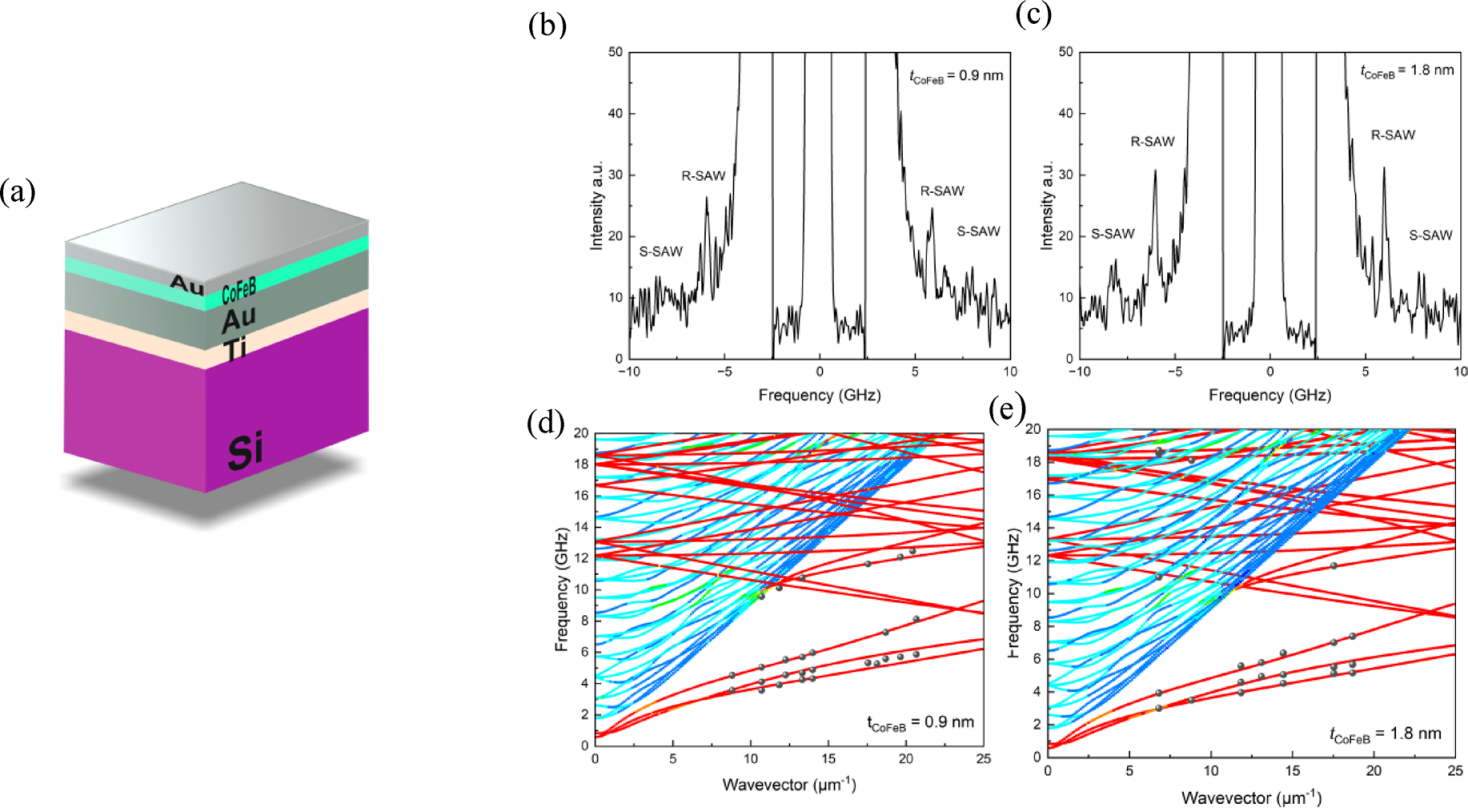
(**a**) Schematic illustration of the investigated multilayer structure. (**b**–**c**) Representative Brillouin spectra showing surface acoustic modes, including Rayleigh (R-SAW) and Sezawa (S-SAW) waves, measured at a wave vector *q* = 20.44 μm^−1^ for two different CoFeB layer thicknesses. (**d**–**e**) Dispersion relations for samples with *t*_CoFeB_= 0.9 nm and 1.8 nm. The black dots indicate experimental data points extracted from the Brillouin spectra, while the color maps represent the simulated acoustic wave modes. Red regions correspond to surface acoustic waves, with visible mode folding, and blue regions indicate bulk acoustic modes.

The elastic properties of different materials can be studied in various ways, including Brillouin spectroscopy. Initially, the focus was on investigating the frequency at which SAWs propagate in systems with varying CoFeB layer thicknesses. Representative Brillouin light scattering (BLS) spectra for the investigated multilayer structures with two selected CoFeB layer thicknesses (0.9 nm and 1.8 nm) are presented in Fig. 1b–c. These spectra clearly reveal the presence of surface acoustic modes, including both Rayleigh surface acoustic waves (R-SAW) and Sezawa modes (S-SAW). The intensity peaks corresponding to these modes are symmetrically distributed around the elastic line, with characteristic frequency shifts up to ~ 8 GHz for *t*_CoFeB_ = 0.9 nm and over 10 GHz for *t*_CoFeB_ = 1.8 nm. The increased frequency shift for the thicker CoFeB layer indicates a significant change in SAW dispersion, which is further analyzed below.

In Fig. 1b–c, a direct comparison of the frequency domain BLS spectra for both samples demonstrates the influence of CoFeB thickness on the position and relative intensity of the acoustic modes. Notably, the Sezawa modes (visible at higher frequencies than R-SAW) become more pronounced as the thickness of the magnetic layer increases. This suggests enhanced elastic contrast and stronger confinement of the acoustic energy in the upper layers, which is consistent with theoretical predictions for multilayers with varying stiffness and density.

Figure 1d–e present the dispersion relations for the same samples, where experimental points (black dots) extracted from the BLS spectra are superimposed on color-coded maps derived from FEM simulations. The color maps reflect the spatial localization of acoustic displacements: red regions correspond to surface-localized modes, while blue regions indicate bulk acoustic wave contributions^32^.

Quantitatively, the experimental dispersion curves demonstrate a clear increase in SAW phase velocity with increasing CoFeB thickness. For example, the phase velocity at *q* = 20 μm^−1^ shifts from approximately 2800 m/s (for *t*_CoFeB_ = 0.9 nm) to over 3200 m/s (for *t*_CoFeB_ = 1.8 nm), which correlates with the expected trend due to the increased stiffness of the effective upper layer. This observation validates the hypothesis that magnetic layer thickness directly influences the elastic properties of the system and, consequently, the propagation dynamics of surface acoustic waves.

Moreover, the good agreement between experimental and simulated data supports the use of the finite element model for predicting acoustic behavior in such complex multilayer systems. Importantly, the comparison also emphasizes the need to consider optical penetration depth in modelling, as only the near-surface region contributes to the BLS signal.

Overall, the results presented in Fig. 1 confirm that even subtle changes in the CoFeB thickness at the sub-nanometer level significantly affect modifications in both the frequency and velocity of SAWs. This sensitivity underscores the potential of SAW-based techniques for high-resolution diagnostics of elastic and magnetoelastic properties in nanoscale layered structures.

Spectra and dispersion relations for different samples with different thicknesses of CoFeB are included in the Supplementary Materials.

## Discussion

The multilayer systems studied are opaque structures with significant variations in elastic parameters and density across the individual layers. The dispersion curves of surface waves are strongly influenced by the nature of the substrate, the composition of the multilayer system, and the specific properties of each layer. In general, the relative velocities of bulk transverse waves allow for the classification of layered systems into two categories: slow-on-fast and fast-on-slow^1,4,8,21,33^. In such systems, surface waves propagate through both the layer and the substrate.

It is important to note that the terms “fast substrate” and “slow layer” refer to the relationship between the velocities of bulk transverse waves propagating in the layer and the substrate. In the slow-on-fast configuration, the presence of a slower layer on a faster substrate reduces the propagation velocity of the surface wave compared to that in the uncoated substrate. Conversely, in the fast-on-slow system, the propagation velocity of SAW increases relative to that in the uncoated substrate, as the bulk transverse wave velocity in the layer exceeds that in the substrate. Thus, a key step in characterizing these systems is determining the relative velocities of bulk waves, particularly transverse waves, within the components of the multilayer structure. This allows for the identification of the system’s classification in terms of acoustic wave propagation. In the examined system, the relative velocities of the slowest bulk transverse waves follow the order: υ_Si_ > υ_CoFeB_ > υ_Ti_ > υ_Au_.

**Fig. 2:**
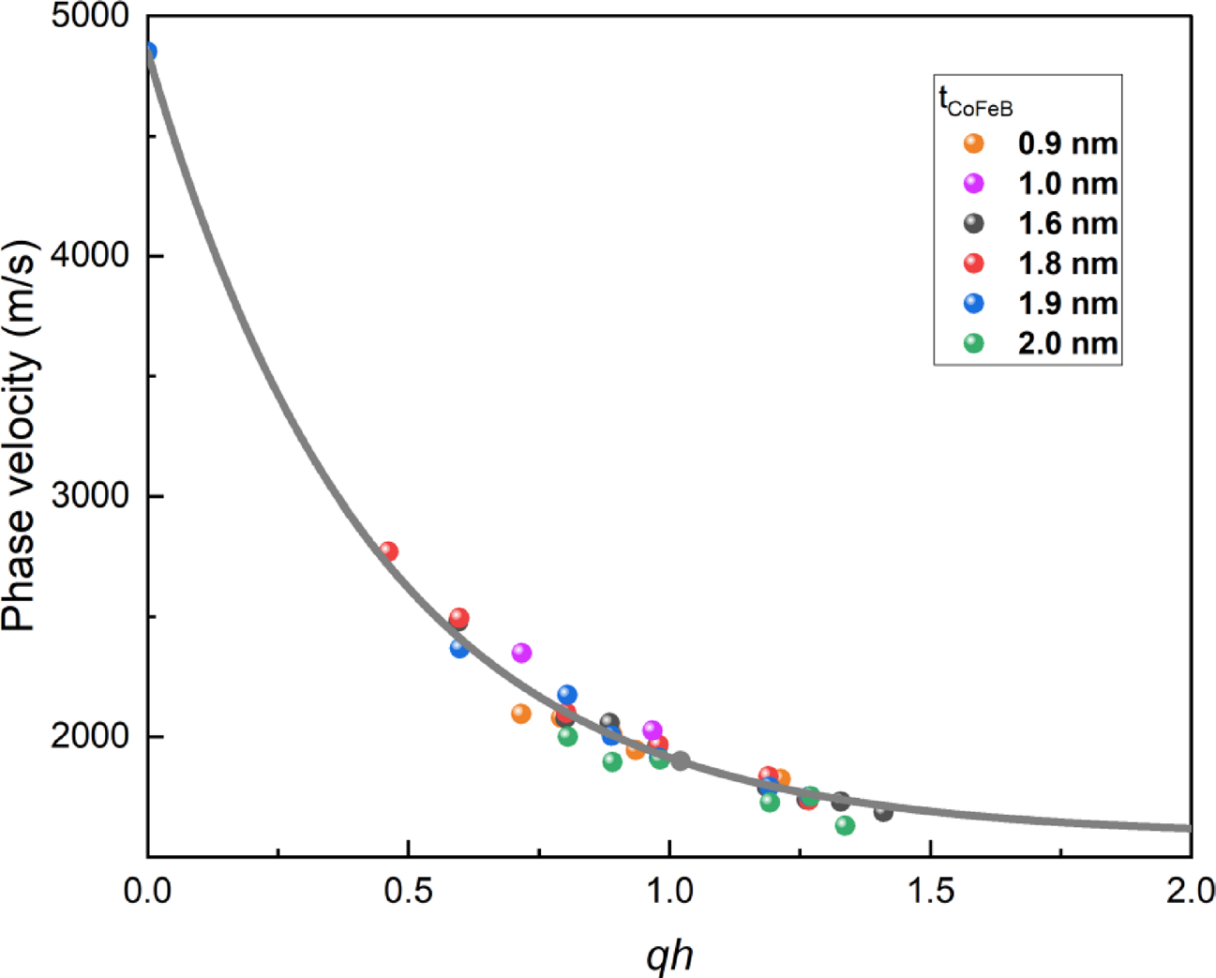
Dependence of the R-SAW phase velocity on the wave number, expressed as the product of the wavevector *q* and the total layer thickness *h* (*h* = *t*_Au_+*t*_CoFeB_+*t*_Ti_). Experimental points are represented by colored spheres.

This clearly indicates that the propagation velocity of waves in each layer is lower than that in the silicon substrate, allowing the system to be preliminarily classified as a slow-on-fast system (Fig. 2).

**Fig. 3:**
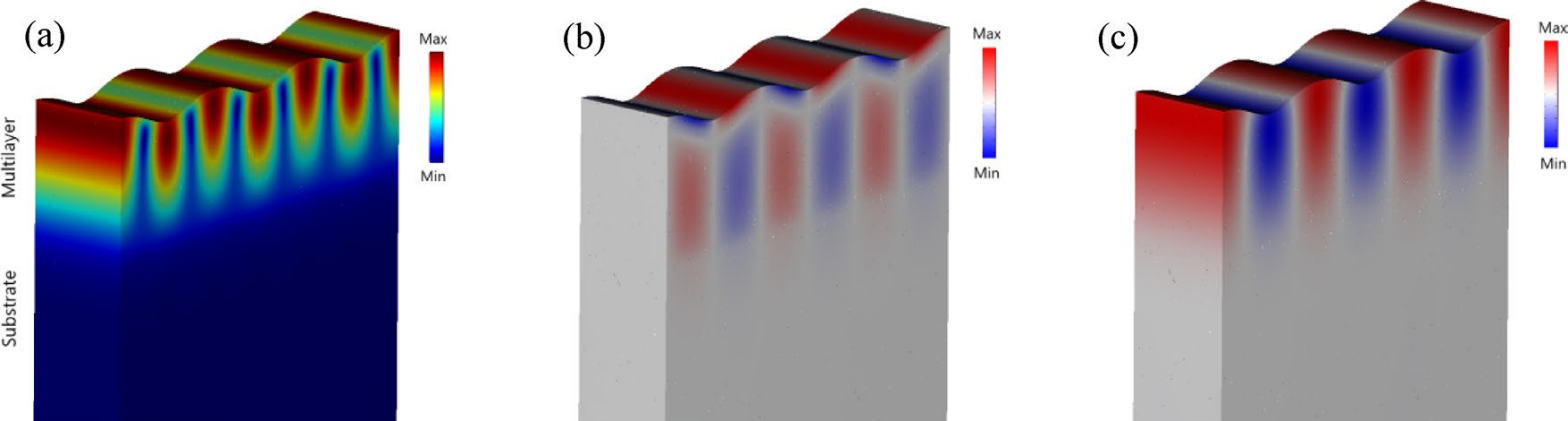
The R-SAW mode profiles show the total displacement (**a**), the *x*-component (**b**) and the *z*-component (**c**) of the displacement for the sample with *t*_CoFeB_ = 0.9 nm.

The analysis of scattering by SAW depends on the characteristics of the sample. In this study, we consider both a semi-infinite, homogeneous medium and thin layers deposited on a substrate. Although interfacial scattering may occur, the surface acoustic waves analyzed here have wavelengths ranging from approximately 250 nm to several micrometers, which are much larger than the thicknesses of the individual layers. As a result, the contribution of interfacial scattering is negligible, justifying the use of the effective medium approximation. This conclusion is further supported by typical surface displacement amplitudes on the order of tens of nanometers^1,34^. Given that the penetration depth of Rayleigh waves is relatively large compared to the thickness of the layers deposited on the silicon substrate, it becomes evident that the deformation caused by SAW propagation extends throughout the entire multilayer system. This phenomenon is illustrated by the wave displacement components shown in the figure below (Fig. 3).

It can be seen that the energy associated with R-SAW propagation is primarily concentrated within the region occupied by the multilayer system. According to simulations, the SAW penetration depth in this structure ranges from approximately 70 nm to several micrometers, depending on the wavevector.

**Fig. 4:**
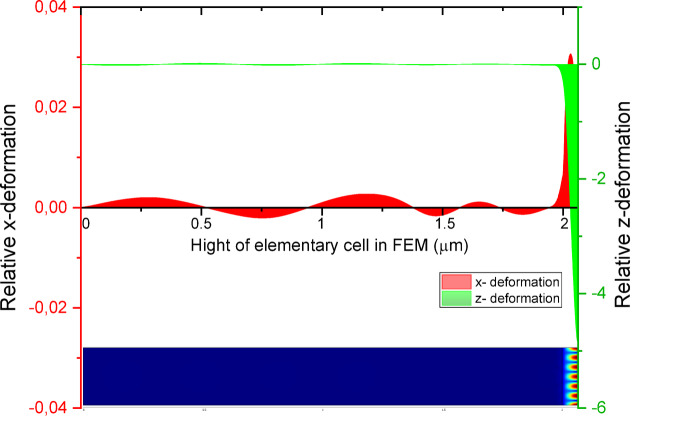
Distribution of relative deformation components for the sample with *t*_CoFeB_ = 0.9 nm:* x*-component (shown in red), *z*-component (shown in green) of deformation, and total relative deformation in the central *YZ*-plane of the unit cell. The term ‘relative deformation’ refers to displacement normalized to the maximum value across the unit cell.

During the FEM simulations, the elementary unit cell used for calculations consisted of a 2 μm thick silicon substrate and a multilayer region ranging from 66 to 86 nm in height, depending on the CoFeB layer thickness. The total height of the unit cell was therefore approximately 2.066–2.086 μm. As shown in Fig. 3, the deformation is indeed concentrated within the multilayer region. To further confirm this observation, the *x*- and *z*-components of the SAW-induced strain were plotted across the vertical axis of the unit cell, as shown in Fig. 4.

It is evident that the relative deformation due to the *x*-component (red line) remains below ± 0.04, while the z-component (green line) reaches values close to 1. These values are normalized with respect to the maximum deformation, allowing for a direct comparison of relative amplitudes. The significantly larger amplitude of the* z*-component clearly indicates that the primary deformation occurs along the vertical axis and decays with depth, as expected for surface acoustic waves (SAWs). For clarity, the total relative deformation field of the unit cell is also shown at the bottom of the figure (in agreement with Fig. 3a), reinforcing the localization of the strain within the multilayer region.

Surface acoustic waves can penetrate materials to a depth of up to twice their wavelength. However, since BLS is an optical technique, the depth from which scattered light is collected is considerably shallower. Therefore, it is essential to first determine the penetration depth of light in the studied multilayer structures.

When light penetrates the sample, its amplitude decreases due to energy dissipation from absorption and scattering. This phenomenon is quantified by the extinction coefficient, which depends on the refractive index and particle size. The extinction coefficient directly influences the depth of material penetration by light waves^21^. This light penetration depth can be calculated using the following formula:

4 $$\delta = \frac{{\lambda _{0} }}{{4\Pi \kappa }}$$

where λ_0_ is the wavelength of the laser (532 nm), and κ is the extension coefficient characteristic for each layer. Table 2 provides information on the refractive index, extinction coefficient, and light wave penetration depth for each layer according to Eq. 4. The effective refractive index, extinction coefficient, and light wave penetration depth for CoFeB were determined based on the chemical composition of the sample, where cobalt, iron, and boron are present in proportions of 20%, 60%, and 20%, respectively.

**Table 2:** Light wave penetration depth in materials present in the Si/Ti/Au/CoFeB/Au sample.

Material	Si	Ti	Au	CoFeB	Au
Refractive index (n)	4.1520	2.4793	0.5439	2.2429	0.5439
Extinction coefficient (κ)	0.0518	3.3511	2.2309	2.5683	2.2309
Light penetration depth (nm)	817	13	19	35	19

Considering the light penetration depth values in individual layers, an approximate method can be applied to determine the overall light penetration depth in the studied system. The average penetration depth for the entire system will be calculated as a weighted average of the penetration depths of each layer, considering the thicknesses of the layers. For each layer, we will multiply the penetration depth by the thickness of the layer, sum these values, and then divide by the total thickness of the system, according to the following equation:

5 $$\delta _{{eff}} = \frac{{\sum \left( {\delta _{n} \cdot t_{n} } \right)}}{{\sum t_{n} }}$$

where $\:{\delta\:}_{n}$ – penetration depth of the n-th layer, $\:{t}_{n}$- thickness of the n-th layer.

Thus, the penetration depth of light in the studied material is approximately 19 nm (for 0.9 nm CoFeB thickness). This directly results from the application of the contribution ratio method based on Table 2. Light will penetrate the system the least when the effective penetration depth is at its minimum, which typically occurs with materials with the highest extinction coefficient (κ) in the first layer through which light passes.

The penetration depth of SAWs in materials can reach up to two SAW wavelengths. However, due to the optical nature of the BLS technique, the actual depth from which the scattered light is collected is significantly smaller. Consequently, treating the multilayer system as an effective layer is valid within the region where light is collected or as one effective layer. A key point of comparison is which modeling approach provides better agreement with experimental data. To answer this, we examine two situations: the first treats multilayers as a single effective layer with effective elastic parameters, while the second approach defines the elastic parameters only in the region where light penetrates.

The effective elastic tensor coefficients $\:{c}_{ij}^{eff}$ for a multilayer system were calculated using the following equation:

6 $$c_{{ij}}^{{eff}} = \frac{1}{h}\mathop \sum \limits_{n} t_{n} c_{{ij}}^{{\left( n \right)}}$$

where $\:{c}_{ij}^{eff}$represents the effective coefficient of the elastic tensor, *t*_*n*_ is the thickness of the n-th layer, $\:{c}_{ij}^{\left(n\right)}$ is the elastic tensor coefficient specific to the n-th layer (presented in Table 1), *h* is the total thickness of the multilayer system, defined as the sum of all individual layer thicknesses:

7 $$h = \mathop \sum \limits_{n} t_{n}$$

The effective density of the multilayer, treated as an effective layer, is calculated using the formula (8):

8 $$\:{\rho\:}_{eff}=\frac{1}{h}\sum\:_{n}{t}_{n}{\rho\:}_{n}$$

For penetration depth, the density is defined according to Eq. (9):

9 $$\rho _{{eff}}^{{pd}} = \frac{{\mathop \sum \nolimits_{n} \delta _{n} \rho _{n} }}{{\mathop \sum \nolimits_{n} \delta _{{eff}} }}$$

where *ρ*_*n*_ is the density of each layer (visible in Table 1), and $\:{\delta\:}_{n}$ is the penetration depth of each layer is presented in Table 2.

Formula 6 essentially calculates a thickness-weighted average of the elastic coefficients across all layers in the system or selected layers. The *c*_*ij*_ ​values are calculated to satisfy the Born stability criteria for elasticity tensors^35^. These are fundamental conditions that must be met for a material to be mechanically stable, ensuring that the strain energy of the material is positively defined. For cubic symmetry, which is indirectly influenced by the substrate symmetry, these conditions are expressed as follows: *c*_11_ > 0, *c*_44_ > 0, *c*_11_ - *c*_12_ > 0, *c*_11_ + 2 *c*_12_ > 0. The density of the effective layer was calculated analogously.

**Fig. 5:**
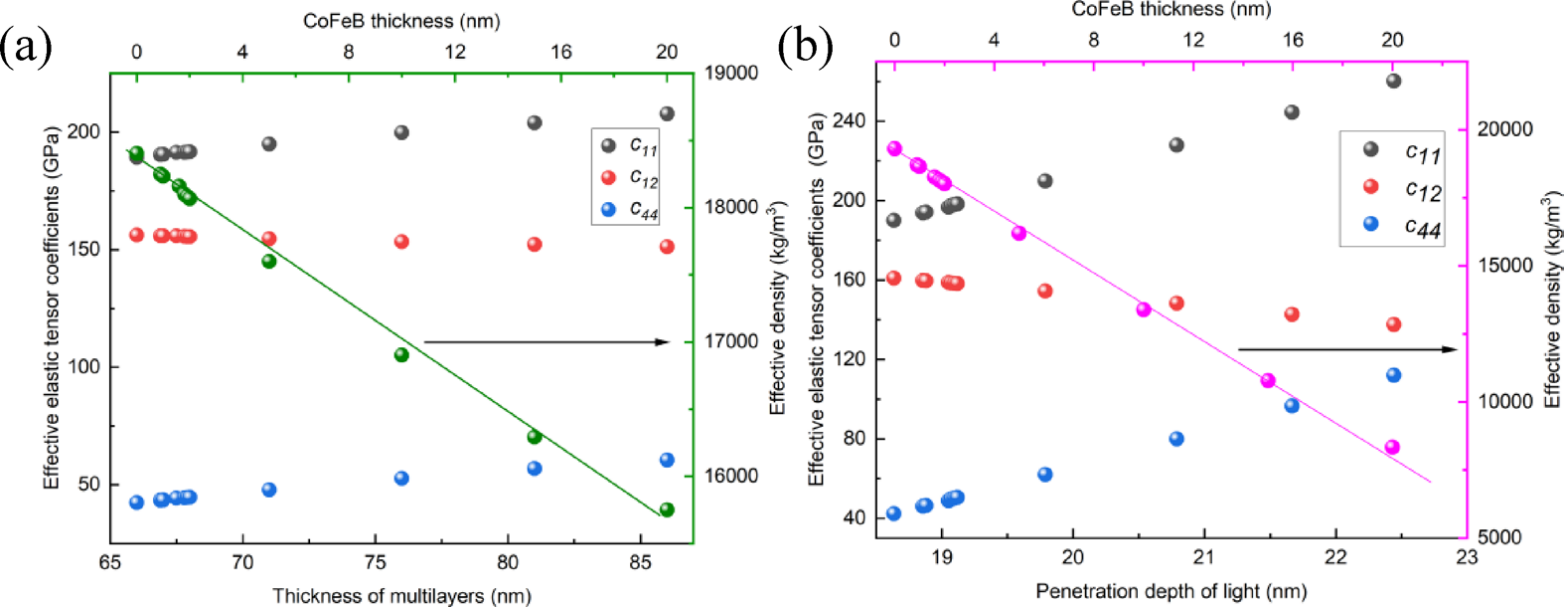
Variation of effective elastic parameters (*c*_11_, *c*_12_, *c*_44_) and density as a function of CoFeB thickness for: (**a**) the entire multilayer system treated as a single effective layer, and (**b**) the region limited by the light penetration depth. The CoFeB thickness range (0–20 nm) is indicated on the top axis. These parameters are used to calculate the SAW dispersion relations under both approaches.

To generalize our observation, we calculated the elastic tensors and densities for CoFeB thicknesses ranging from 0 nm to 20 nm in the studied samples (Fig. 5). The change in CoFeB thickness affects the calculated parameters when treating the multilayer system as a single effective layer. Depending on the penetration depth of the light, the values of the elastic tensor and density also vary. It illustrates that the values of *c*_*ij*_ and density are more sensitive to changes in the CoFeB thickness when only considering the thickness of the region penetrated by light.

The individual layers in the multilayer structure differ significantly in both density and the values of the elastic tensor components. The gold layer exhibits the highest values in this regard (see Table 1). Applying the effective medium approach described by Eqs. (6)–(9) results in each layer’s contribution to the global parameters being weighted according to its thickness. When the entire multilayer stack is treated as a single effective layer, a change in the CoFeB layer thickness leads to only minor variations in the elastic properties and density, as the CoFeB contribution to the total thickness of the system remains relatively small.

In contrast, when the effective parameters are determined only for the region corresponding to the light penetration depth, only the CoFeB and Au layers are considered in the analysis. In this approach, even a small change in CoFeB thickness significantly affects the effective parameters, as it noticeably alters the thickness ratio between the CoFeB and gold layers.

**Fig. 6:**
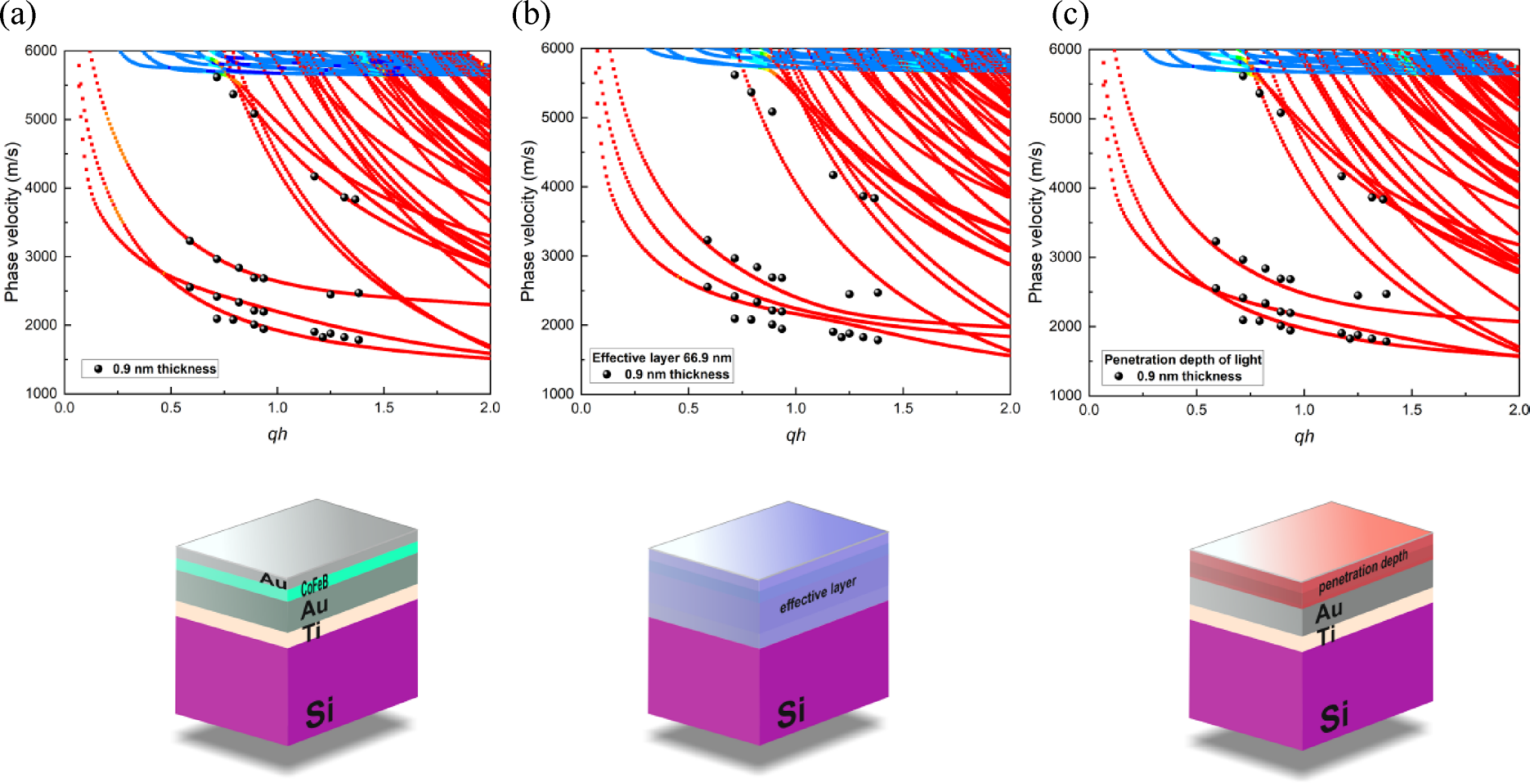
Comparison of experimental and simulated SAW phase velocity dispersion for S/Ti/Au/CoFeB (*t*_CoFeB_ = 0.9 nm)/Au sample as a function of wave number. Three modeling approaches are presented: (**a**) full multilayer treatment with distinct properties for each layer, (**b**) complete system treated as a single effective layer with thickness-weighted elastic parameters (blue region in schematic), and (**c**) effective layer limited to the light penetration depth of 19 nm (red region in schematic). Black points represent experimental BLS measurements, while colored points show FEM simulation results using parameters from Table 1; Fig. 5.

By utilizing the elastic parameters and density presented in Table 1; Fig. 5, we calculate the phase velocity of the SAW for a sample with 0.9 nm of CoFeB, as shown in Fig. 6.

The agreement between simulation and experimental results is better when the multilayer system is treated as an effective layer within the light penetration region rather than as a single effective layer (Fig. 6b and c). This approach demonstrates that the capping layers have a significant impact on SAW propagation velocity. However, it is essential to consider the light penetration depth within the given multilayer system (Fig. 6c). A comparison of different approaches indicates that, even when treating the system as a multilayer, it can still be regarded as an effective layer within the light penetration region. This becomes evident when comparing the agreement between simulations and experimental results in the figure above. Notably, the poorest fit occurs when the entire multilayer system is treated as a single effective layer (Fig. 6b).

**Fig. 7:**
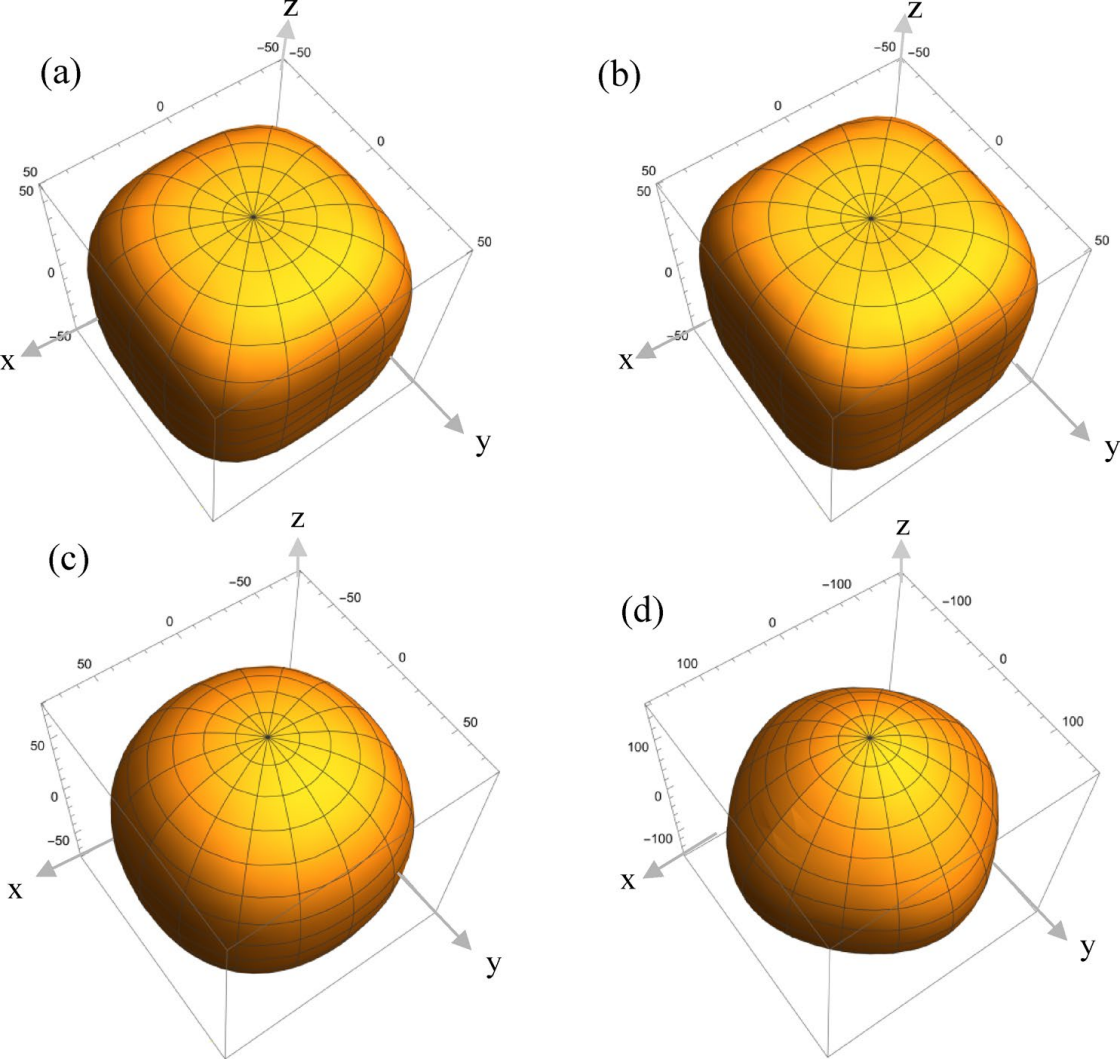
The 3D view of the calculated Young’s modulus [GPa] for different CoFeB thicknesses in the sample: *t*_CoFeB_ = 0.9 nm (**a**, **b**) and *t*_CoFeB_ = 20 nm (**c**, **d**) treated as an effective layer (**a**, **c**) and only within the penetration depth region (**b**, **d**).

For the studied systems, Young’s modulus was determined in two different scenarios (Fig. 7). All calculations were performed using Eqs. 10–13 in the first approach for the multilayer system treated as a single effective layer. In the second approach, the effective layer was considered only within the laser light penetration region—approximately 19 nm for the sample with a 0.9 nm CoFeB layer thickness. Young’s modulus, *E*, can be calculated using the following equation^36,37^:

10 $$E = \frac{1}{{s_{{33}}^{\prime} }}$$

.where *s’*_33_ is the stiffness component in the system’s measurement plane (expressed in Voigt notation). For a material with cubic symmetry, this component is defined as follows^38^ :

11 $$s_{{33}}^{\prime} = s_{{11}} - \left( {s_{{11}} - s_{{12}} - \frac{1}{2}s_{{44}} } \right)\left[ {1 - \cos ^{4} \varphi \sin ^{4} \psi - \sin ^{4} \varphi \sin ^{4} \psi - \cos ^{4} \psi } \right]$$

where *s*_11_, *s*_12_, *s*_44_ represent the stiffness components in the system’s crystal plane, while *ϕ* and *ψ* are arbitrary rotation angles used to transform the cubic crystal coordinate system into a lattice plane system. The relationships between the stiffness components *s*_11_, *s*_12_, *s*_44_ and the elastic constants *c*_11_, *c*_*12*_, *c*_44_ are given as follows:

$${s}_{11}=\frac{{c}_{11}+{c}_{12}}{\left({c}_{11}-{c}_{12}\right)\left({c}_{11}+{2c}_{12}\right)\:}$$

12 $${s}_{12}=\frac{-{c}_{12}}{\left({c}_{11}-{c}_{12}\right)\left({c}_{11}+{2c}_{12}\right)}$$

$${s}_{44}=\frac{1}{{c}_{44}}$$

By applying Eqs. (10)–(12), Young’s modulus *E* can be expressed as:

13 $$E = \frac{{2\left( {c_{{11}} - c_{{12}} } \right)\left( {c_{{11}} + 2c_{{12}} } \right)c_{{44}} }}{{2\left( {c_{{11}} + c_{{12}} } \right) - \left( {c_{{11}} + 2c_{{12}} } \right)\left( {2c_{{44}} - c_{{11}} + c_{{12}} } \right)\left[ {1 - \cos ^{4} \varphi \sin ^{4} \psi - \sin ^{4} \varphi \sin ^{4} \psi - \cos ^{4} \psi } \right]}}$$

This equation is applicable to a cubic crystal system and considers the variation of Young’s modulus (*E*) with respect to the wave propagation direction within the crystal. Additionally, we utilized ELATE^39 ^an open-source online tool for elastic tensor analysis, to derive Young’s modulus from the given elastic tensor.

As seen in Fig. 6, the anisotropy of the Young’s modulus changes with increasing CoFeB thickness. This effect is observed both when treating the multilayer system as a single effective system and when calculating the Young’s modulus within the light penetration region. The variation in Young’s modulus with respect to the crystallographic direction can be determined using the elastic anisotropy of the crystalline material, known as Zener anisotropy (A). Zener anisotropy for cubic structure can be calculated by the equation^38,40–43^ :

14 $$A = \frac{{2c_{{44}} }}{{c_{{11}} - c_{{12}} }}$$

where *c*_11_, *c*_12_ and *c*_44_ are the elastic constants. The results obtained for the elastic parameters and Young’s modulus are presented in Table 3.

**Table 3:** The Zener anisotropy values (A) for the samples with different cofeb thicknesses, considering both for effective layer and light penetration depth.

CoFeB thickness (nm)	Thickness of effective layer (nm)	A	Penetration depth of light (nm)	A
0	66	2.56	18.64	2.92
0.9	66.9	2.52	18.86	2.71
1	67	2.51	18.88	2.69
2	68	2.47	19.12	2.52
5	71	2.38	19.79	2.24
10	76	2.27	20.79	2.01
15	81	2.19	21.67	1.90
20	86	2.14	22.44	1.83

From an application perspective, these studies are significant as they enable the control of surface wave frequency by adjusting the thickness of the magnetic component in the layered material. This allows for precise tuning of the elastic and dynamic properties of multilayer structures, which is crucial for modern acousto-electronic and spintronic technologies. The ability to manipulate the interaction between acoustic and spin waves through structural modifications has significant implications for next-generation communication and computing technologies. For example, controlling SAW frequency enables optimization of resonators and surface wave filters, which are fundamental components in RF (radio frequency) and microwave devices used in wireless communication, radar systems, and high-frequency signal processing^44^. Additionally, the ability to adjust the thickness of effective layers in a multilayer structure affects the penetration depth of SAWs, determining how much of the wave energy is confined to the surface or distributed deeper into the material. This aspect is critical for enhancing wave confinement, minimizing energy loss, and improving device sensitivity^45^. Ultimately, these studies not only contribute to the fundamental understanding of SAW behavior in complex layered materials but also drive innovations in advanced acousto-electronic and spintronic devices, paving the way for more efficient and compact technologies in telecommunications, sensing, and computing.

A parallel line of research emphasizes that thickness-dependent effects are equally crucial in ultrathin magnetic layers such as CoFeB, where both magnetic and elastic (magnetoelastic) anisotropies are strongly influenced by the interplay between surface and volume contributions. It is well-established that varying the thickness of CoFeB layers leads to a nonlinear dependence of perpendicular magnetic anisotropy (PMA), a phenomenon that has been both experimentally confirmed and theoretically modelled. Recent studies suggest that these magnetoelastic effects are intrinsic to such systems, making independent tuning of magnetic and elastic properties challenging, yet representative of realistic device fabrication conditions^5^. The concept of an “effective layer” based on light penetration depth has proven useful in interpreting optical and magneto-optical measurements, highlighting how device responses can vary spatially within the layer due to finite excitation or probing depth. Although this modelling approach does not directly reflect full layer switching during actual device operation, it provides valuable insight into the spatial distribution of magnetic and elastic properties. Despite these complexities, ultrathin CoFeB layers with strong PMA remain highly promising for memory applications, as demonstrated by recent successes in achieving thermally stable, high-speed switching in sub-nanometer CoFeB/MgO structures^1,4^. Continued efforts in material engineering aim to decouple or jointly optimize these anisotropies, further advancing the design of efficient, high-performance spintronic and magnetoacoustic devices^2,3^.

## Conclusions

The study reveals the critical influence of CoFeB layer thickness on surface acoustic wave (SAW) propagation characteristics. By examining variations in layer thickness from 0.9 nm to 2 nm, we observed significant changes in the propagation of Rayleigh (R-SAW) and Sezawa (S-SAW) waves, highlighting the sensitivity of acoustic wave behavior to nanoscale structural modifications. Light penetration depth analysis demonstrated that accurate modelling requires consideration of only an approximately 19 nm thick layer. This insight significantly improves the precision of effective medium modelling, addressing a crucial limitation in previous experimental approaches. Neglecting light penetration depth can lead to inaccurate estimations of acoustic wave velocities, emphasizing the importance of considering this parameter for obtaining reliable and reproducible results.

The Zener anisotropy coefficient of the studied samples exhibits a systematic decrease with increasing CoFeB layer thickness. This trend indicates a progressive homogenization of the material’s elastic properties and confirms the moderate elastic anisotropy of CoFeB, providing deeper insights into the material’s structural evolution at the nanoscale.

The proposed methodology, which integrates finite element method numerical simulations with Brillouin light scattering experimental measurements, offers a robust and precise toolkit for predicting acoustic wave behavior in complex multilayered structures. This approach represents a significant advancement in characterizing advanced material systems. This phenomenon opens novel opportunities for manipulating acoustic wave properties in spintronic and acoustic devices, where precise control of wave propagation is paramount.

## Electronic supplementary material

Below is the link to the electronic supplementary material.

Supplementary Material 1

## Data Availability

Data availability statement The authors declare that the data supporting the findings of this study are available within the paper, its supplementary information files.
