# Supplementary material for: Influence of effective thickness in elastic anisotropy and surface acoustic wave propagation in CoFeB/Au multilayer
Source: Sci Rep. 2025 Jul 15;15:25585. doi: 10.1038/s41598-025-08560-8 (PMC12264179; doi:10.1038/s41598-025-08560-8)
Supplement: Supplementary file 1 — Supplementary Material 1 [file 41598_2025_8560_MOESM1_ESM.docx]

**Supplementary for Influence of effective thickness in elastic anisotropy and surface acoustic wave propagation in CoFeB/Au multilayer**

A. V. Achuthan^1^, S. Janardhanan^1^, P. Kuświk^2^, A. Trzaskowska^1*^

*^1^ ISQI, Faculty of Physics and Astronomy, Adam Mickiewicz University, Uniwersytetu Poznańskiego 2, 61-614 Poznan, Poland*

*^2^ Institute of Molecular Physics, Polish Academy of Science, Smoluchowskiego 17, 60-179 Poznan, Poland.*

* E-mail: olatrzas@amu.edu.pl

**S1. Brillouin spectra of the material**

Surface acoustic waves investigated in the Si/Ti/Au/CoFeB/Au multilayer sample arise naturally from thermal lattice vibrations, commonly referred to as thermal phonons. These phonons are randomly generated due to thermal motion of the lattice atoms.

Brillouin Light Scattering (BLS) uses coherent laser light with a wavelength of λ₀ = 532 nm to probe these thermally excited phonons. When the laser light interacts with the vibrating lattice, part of it is inelastically scattered. Although thermal phonons are inherently incoherent and propagate in random directions, only those phonons that fulfill energy and momentum conservation conditions—i.e., those with specific frequencies and wave vectors matching the geometry of incident and scattered light—contribute to the detectable Brillouin signal [S1–S3]. As a result, sharp spectral peaks corresponding to surface acoustic modes (such as Rayleigh and Sezawa waves) can be observed. Figure S1A shows the schematic configuration of the BLS experiment. The scattering wave vector *q* lies in the sample plane and defines the direction of phonon detection. Its magnitude increases with the angle of incidence of the probing laser light. Phonons are detected only along the specific direction defined by q, which is calculated using the following expression:

*q*$=\frac{4\pi sinϴ}{\lambda_{0}}$ (S1)

where Θ is the angle of incidence of the incoming photons and *λ_0_* is the wavelength of the laser light.


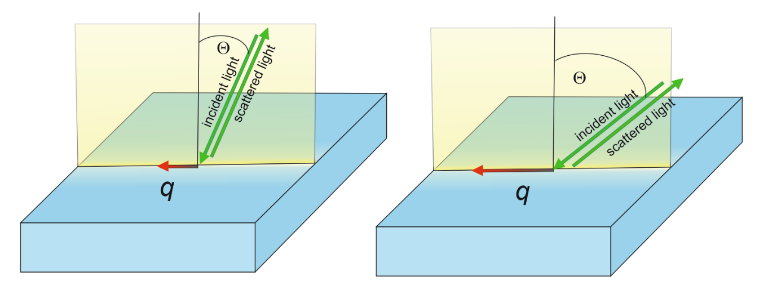


Figure S1A: Schematic diagram of the light interaction with the sample with different angle of incident light Θ and wavevector *q*.

(1)

Fig. S1B shows the Brillouin spectra of the studied materials, presenting surface acoustic modes, including Rayleigh (R-SAW) and Sezawa (S-SAW) waves, for *q* = 20.44 μm^-1^ for various CoFeB layer thicknesses in the sample Si/ Ti(4 nm)/Au(60 nm)/CoFeB(*t*_CoFeB_)/Au(2 nm).


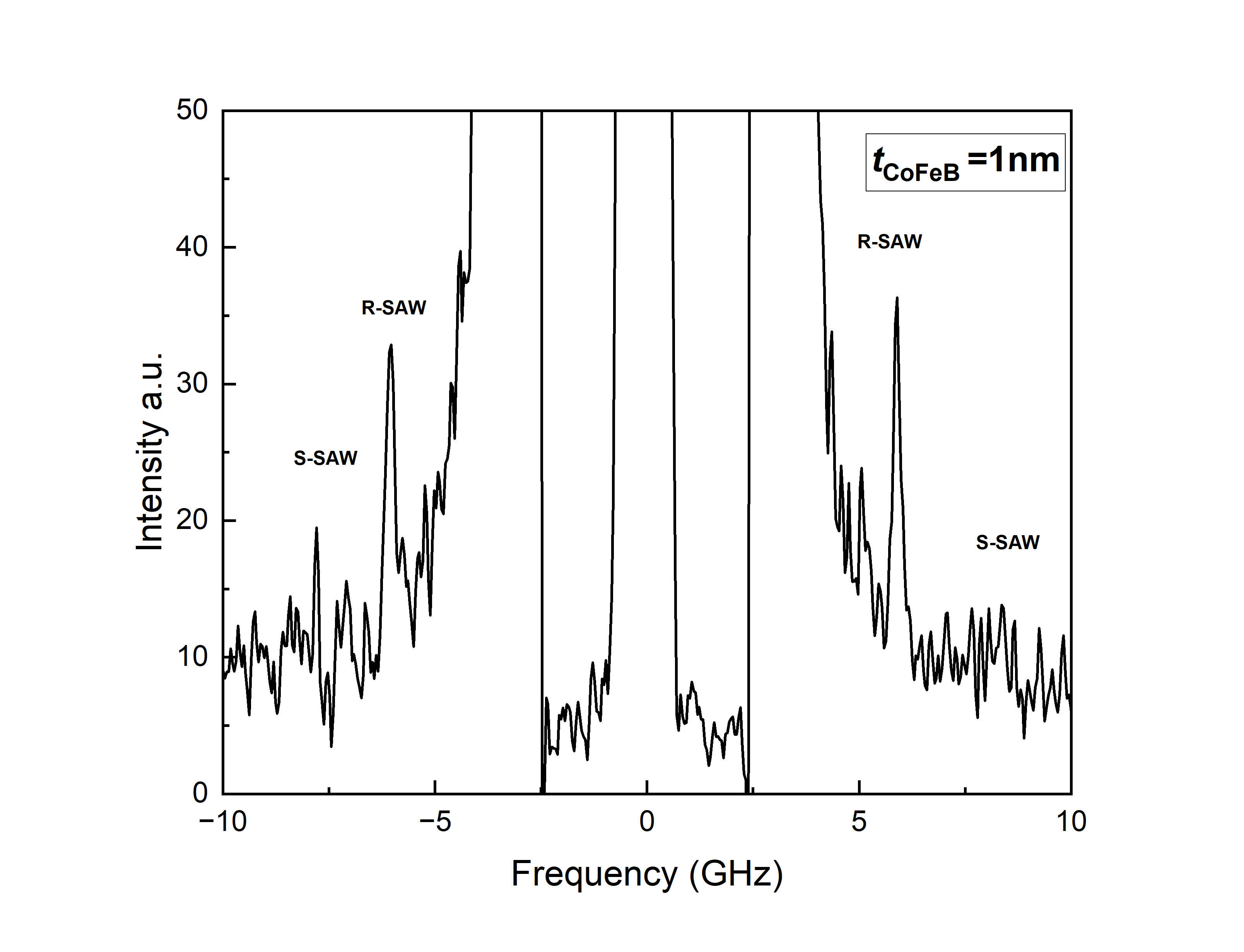

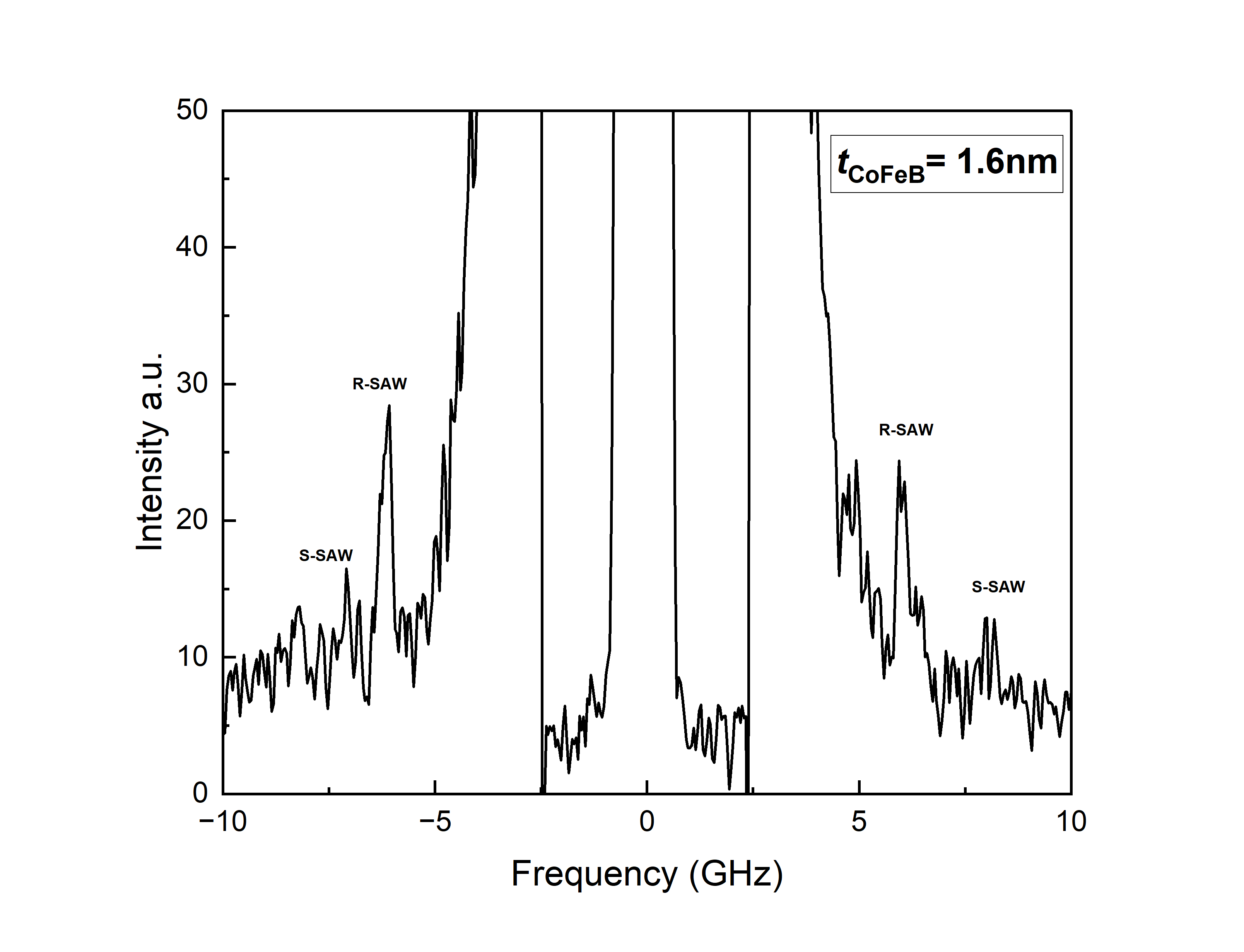

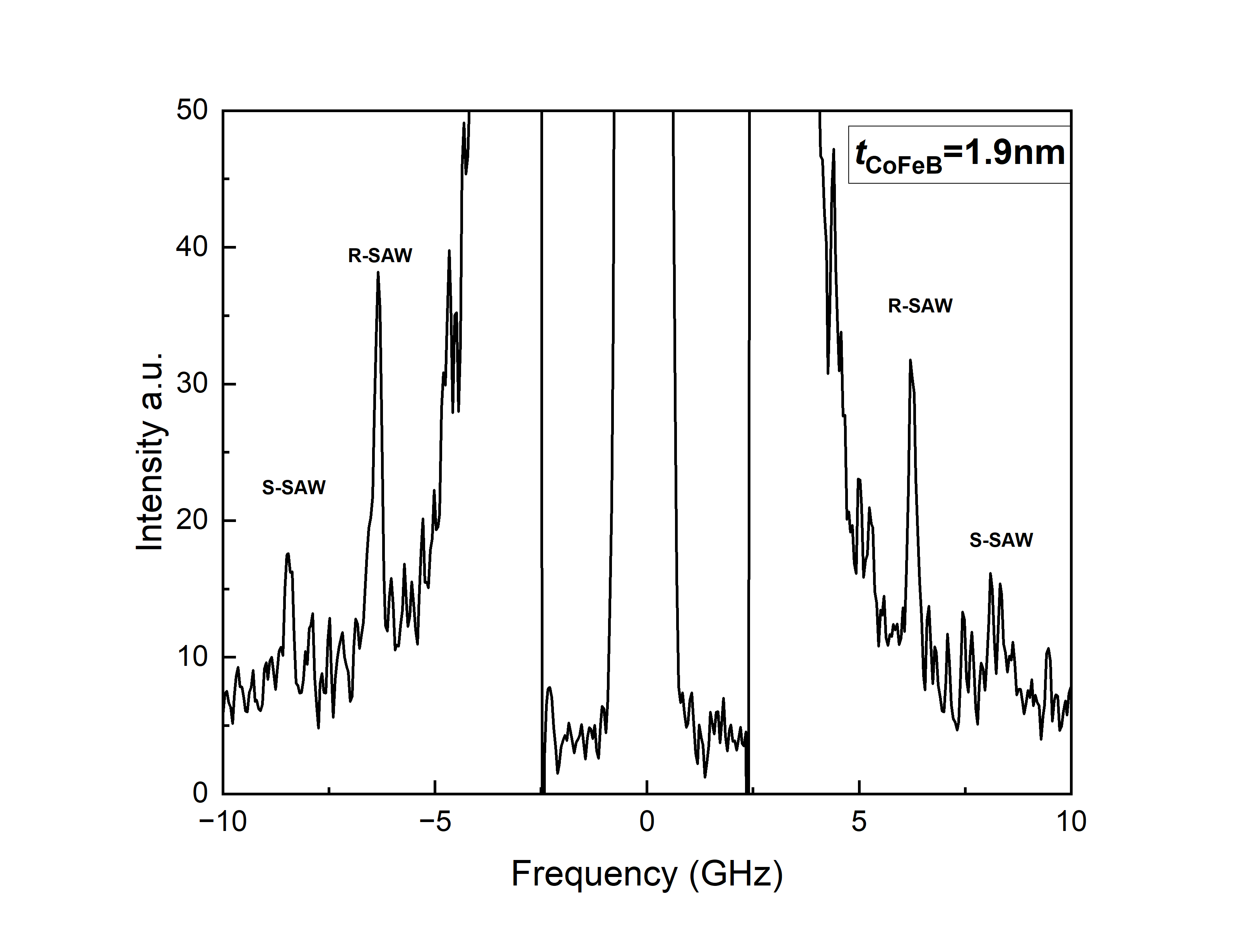

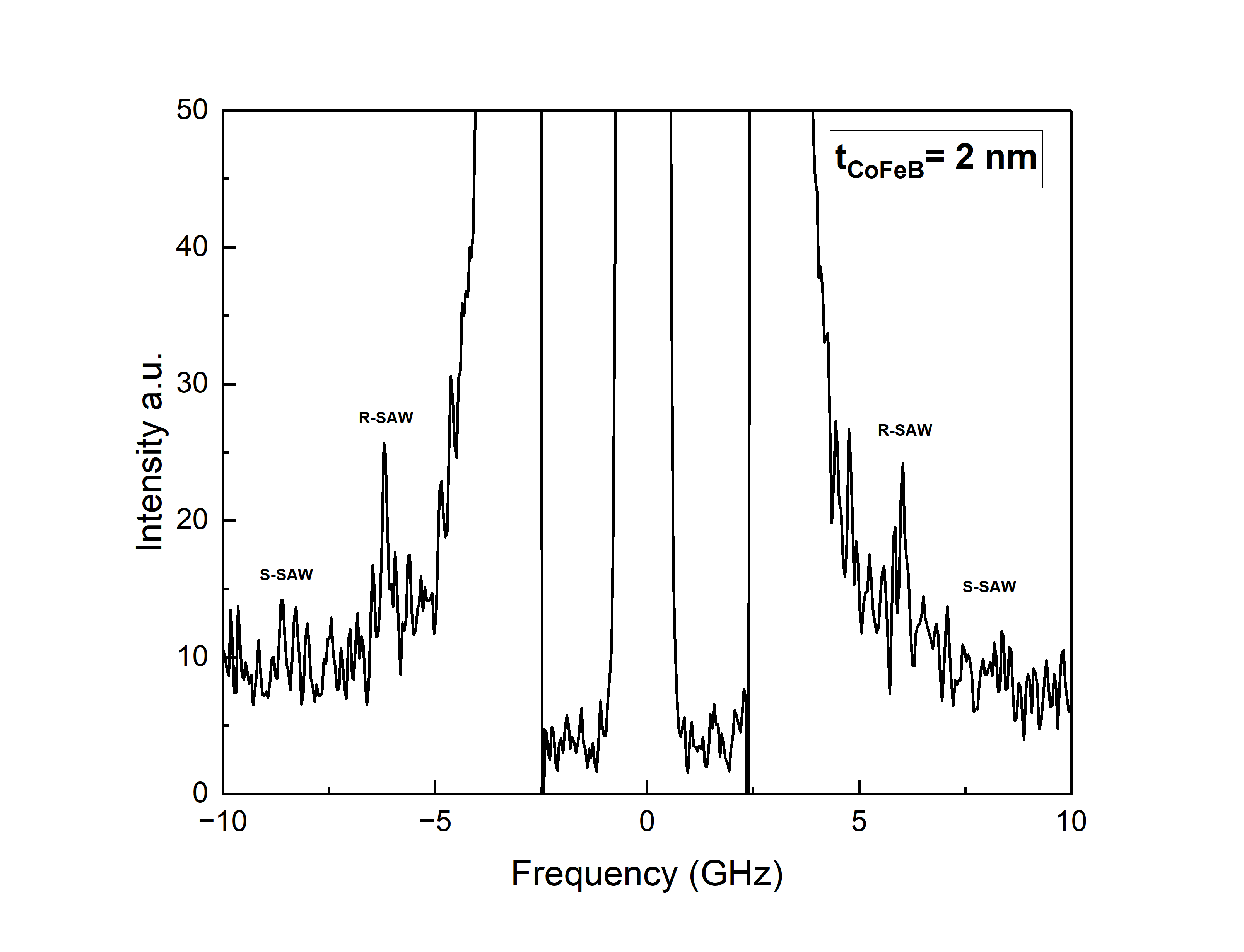


(a)

(c)

(d)

(b)

Fig. S1B. The Brillouin spectra for different thicknesses of CoFeB ((a) *t*_CoFeB_ = 1 nm, (b) *t*_CoFeB_ = 1.6 nm, (c) *t*_CoFeB_ = 1.9 nm and (d) *t*_CoFeB_ = 2 nm respectively) in the samples studied.

**S2. Dispersion relation of phonon**

The comparison between phonon dispersion relations obtained from experimental measurements and simulated data is shown in Fig. S2. The black dots represent the experimental data, whereas the colour map represents the simulated high-intensity data.


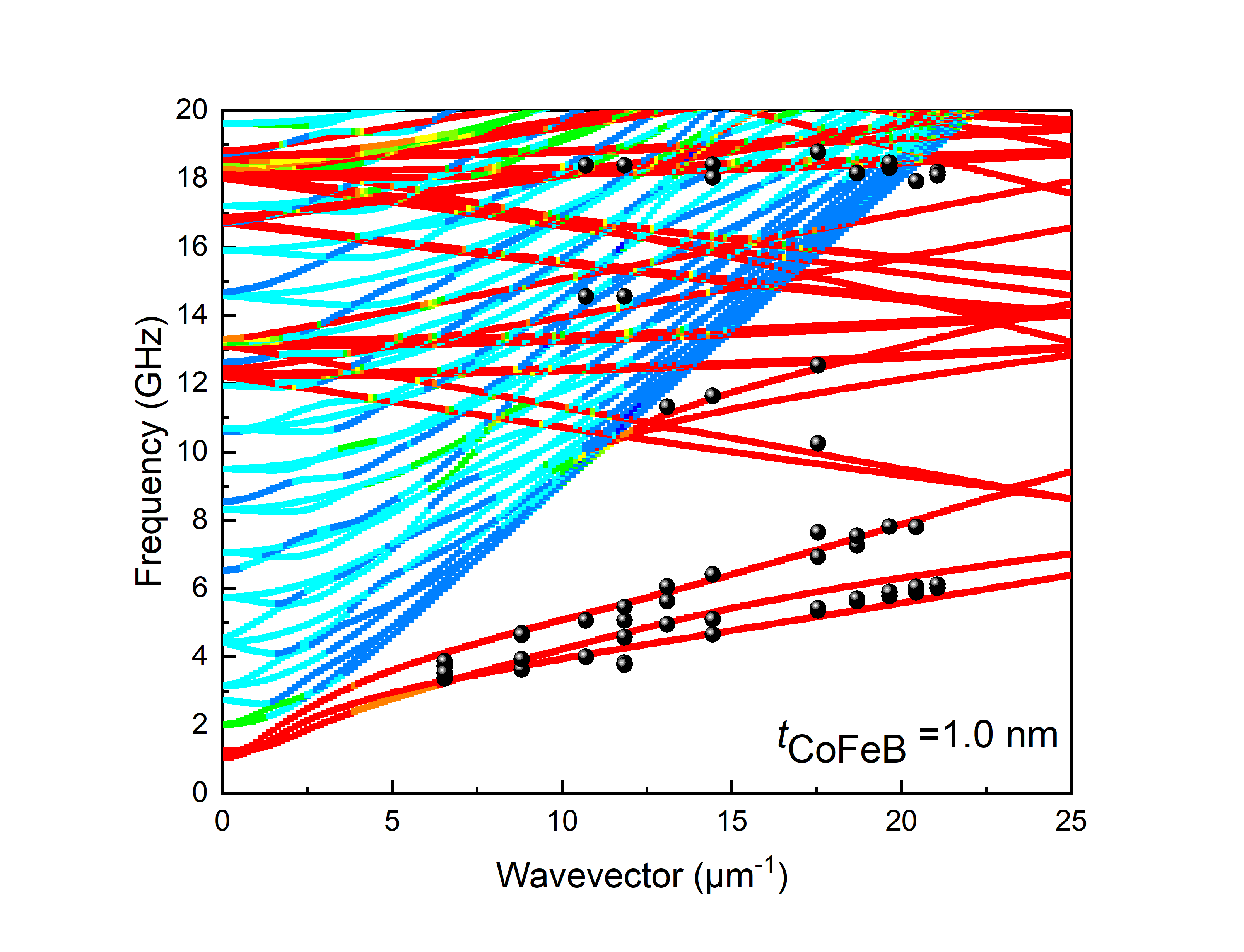

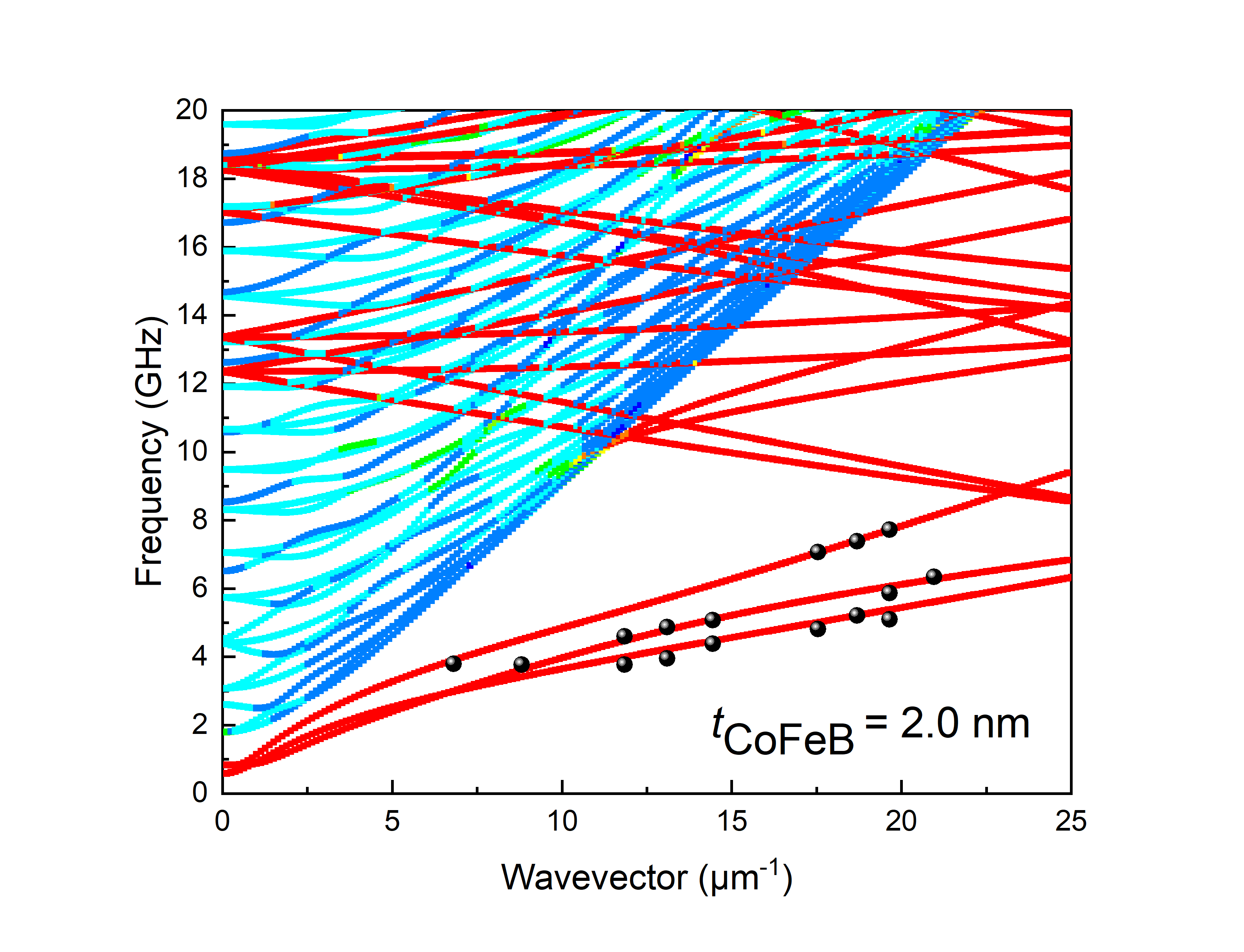


(b)

(a)

Fig. S2. The dispersion relation of phonons for the sample with CoFeB thicknesses of (a) *t*_CoFeB_ = 1 nm and (b) *t*_CoFeB_ = 2 nm. Black dots represent studied data, and the colour map represents simulated data*.*

**S3.** **Effective elastic parameters and density**

The calculated values for the individual CoFeB layer thicknesses are presented in the table below.

Table S1. Effective elastic parameters and density for the multilayers are treated as a single effective layer, and for the case where the multilayer is considered effective in the region of light penetration.

|  | Effective layered system | | | | | Penetration depth system | | | | |
| --- | --- | --- | --- | --- | --- | --- | --- | --- | --- | --- |
|  |  | Elastic tensor | | |  |  | Elastic tensor | | |  |
| CoFeB thickness | thickness | *c_11_* | *c_12_* | *c_44_* | density | Penetration depth | *c_11_* | *c_12_* | *c_44_* | density |
| 0 | 66 | 189.27 | 156.15 | 42.37 | 18403 | 18.64 | 190 | 161 | 42.30 | 19300 |
| 0.9 | 66.9 | 190.34 | 155.85 | 43.42 | 18245 | 18.86 | 193.76 | 159.74 | 46.04 | 18713 |
| 1 | 67 | 190.46 | 155.82 | 43.53 | 18233 | 18.88 | 194.17 | 159.61 | 46.45 | 18649 |
| 1.6 | 67.5 | 191.44 | 155.87 | 44.28 | 18160 | 19.05 | 196.62 | 158.79 | 48.89 | 18267 |
| 1.8 | 67.8 | 191.38 | 155.58 | 44.44 | 18100 | 19.07 | 197.44 | 158.52 | 49.69 | 18139 |
| 1.9 | 67.9 | 191.49 | 155.54 | 44.56 | 18084 | 19.09 | 197.84 | 158.38 | 50.09 | 18076 |
| 2 | 68 | 191.61 | 155.51 | 44.67 | 18068 | 19.12 | 198.24 | 158.25 | 50.49 | 18013 |
| 5 | 71 | 194.87 | 154.63 | 47.88 | 17600 | 19.79 | 209.91 | 154.35 | 62.08 | 16192 |
| 10 | 76 | 199.74 | 153.32 | 52.66 | 16903 | 20.79 | 227.90 | 148.35 | 79.96 | 13384 |
| 15 | 81 | 204 | 152.17 | 56.85 | 16292 | 21.67 | 244.55 | 142.79 | 96.50 | 10785 |
| 20 | 86 | 207.77 | 151.16 | 60.56 | 15751 | 22.44 | 260.23 | 137.56 | 112.08 | 8338 |

**S4. Comparison of simulation data**

Figure S3 presents a comparison between experimental and simulated phase velocity dispersion of SAWs for the S/Ti/Au/CoFeB(*t*_CoFeB_ = 1.8 nm)/Au sample as a function of wave number (a-c) and differences between results obtained from simulation for higher values of CoFeB thickness d-l. Three different modelling approaches are considered. In the first approach, the system is treated as a full multilayer structure, with each layer assigned distinct elastic properties. The second approach models the entire multilayer stack as a single effective medium, using elastic parameters weighted by the thickness of each layer. The third approach further simplifies the model by limiting the effective medium to the optical penetration depth in the range of 19 - 22 nm. Experimental data obtained from Brillouin light scattering (BLS) measurements are shown as black points.

c

b

a


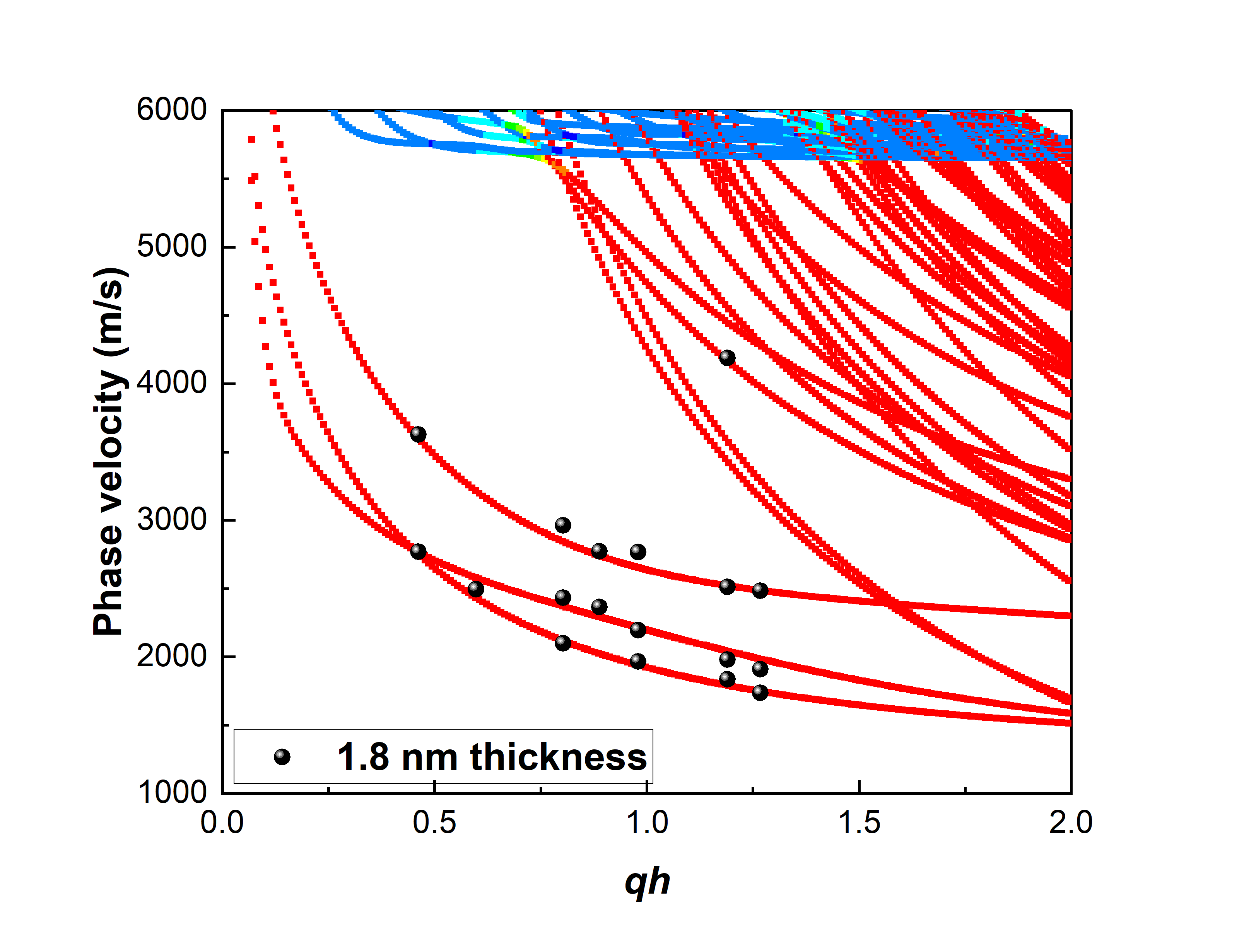

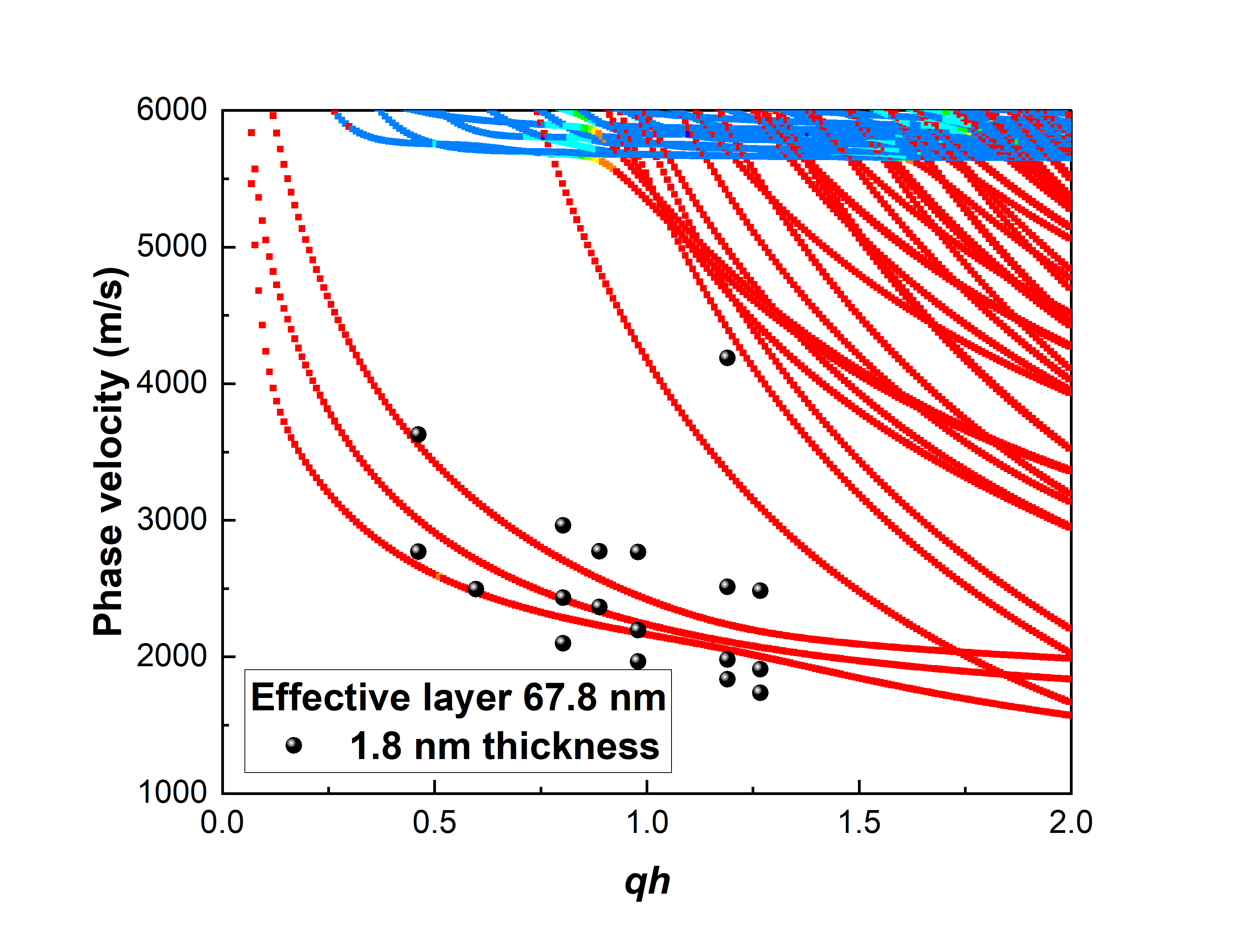

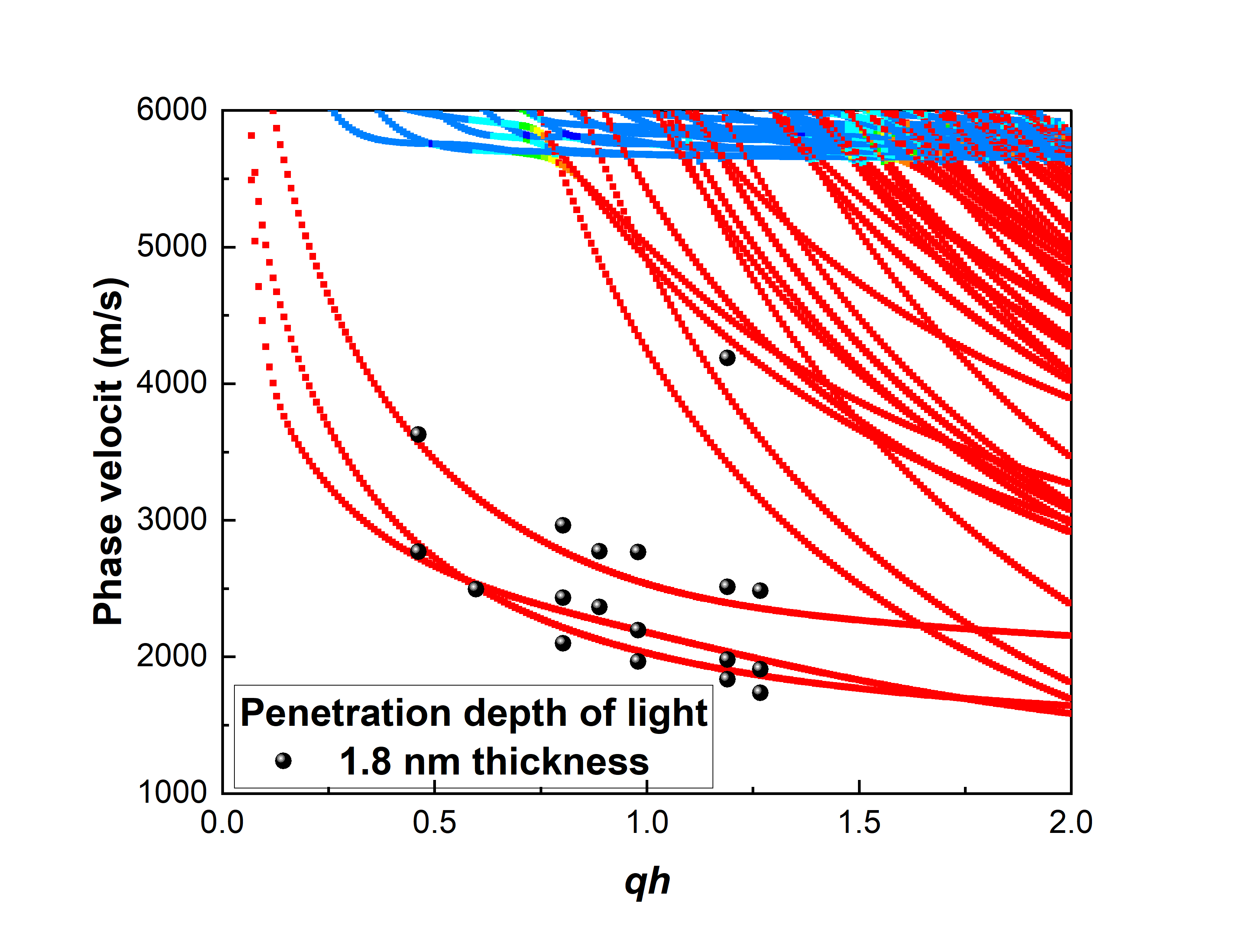


e

d

f


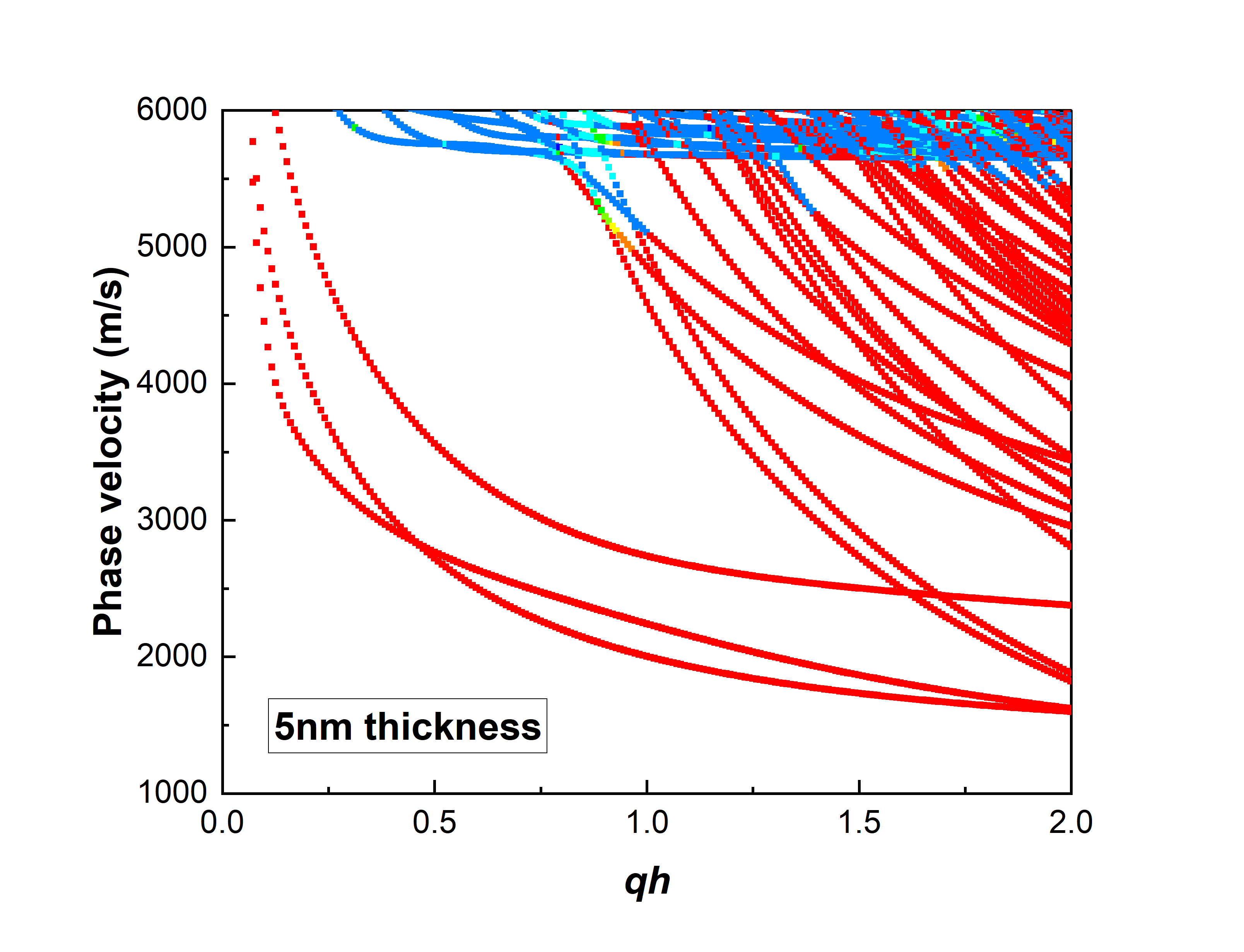

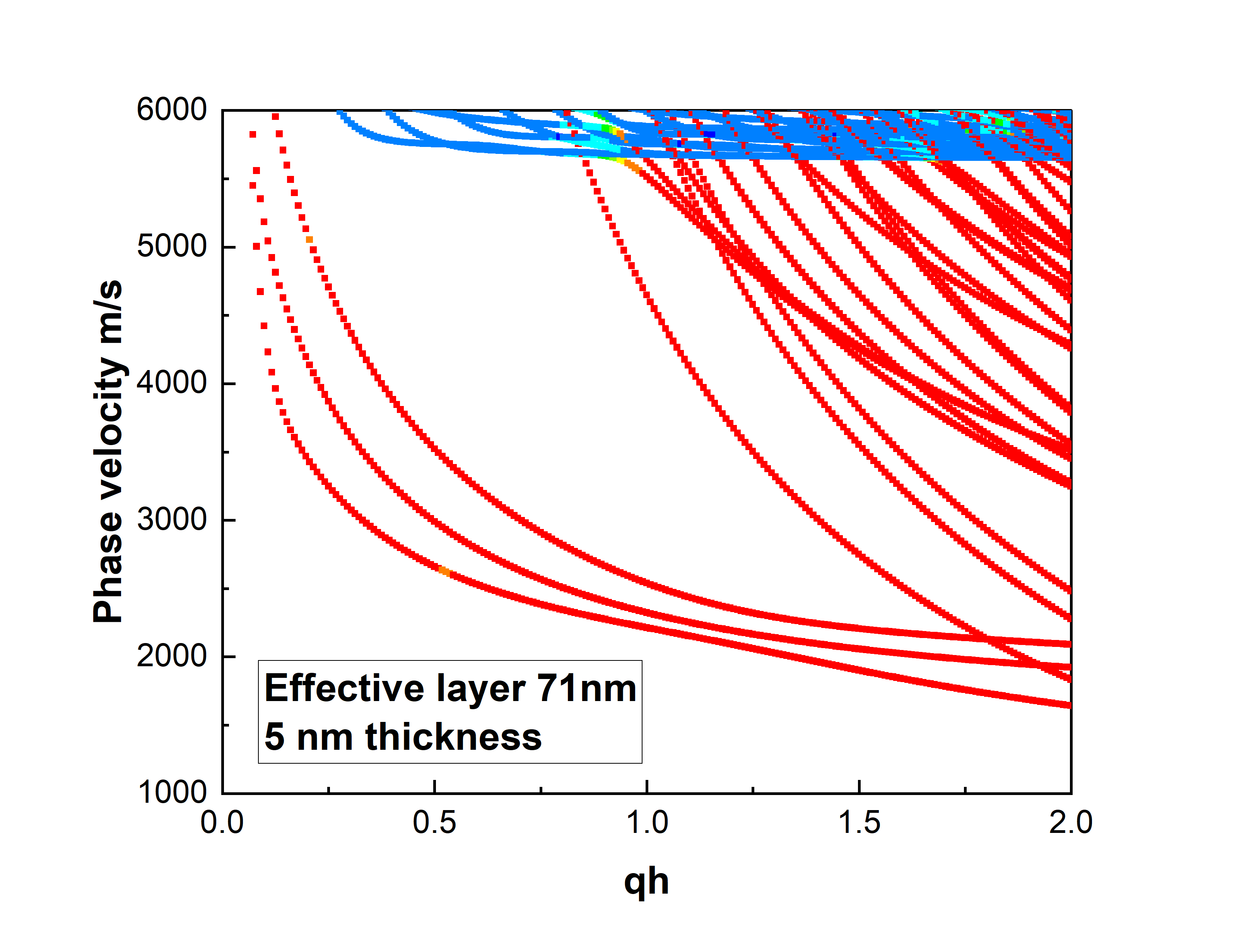
**
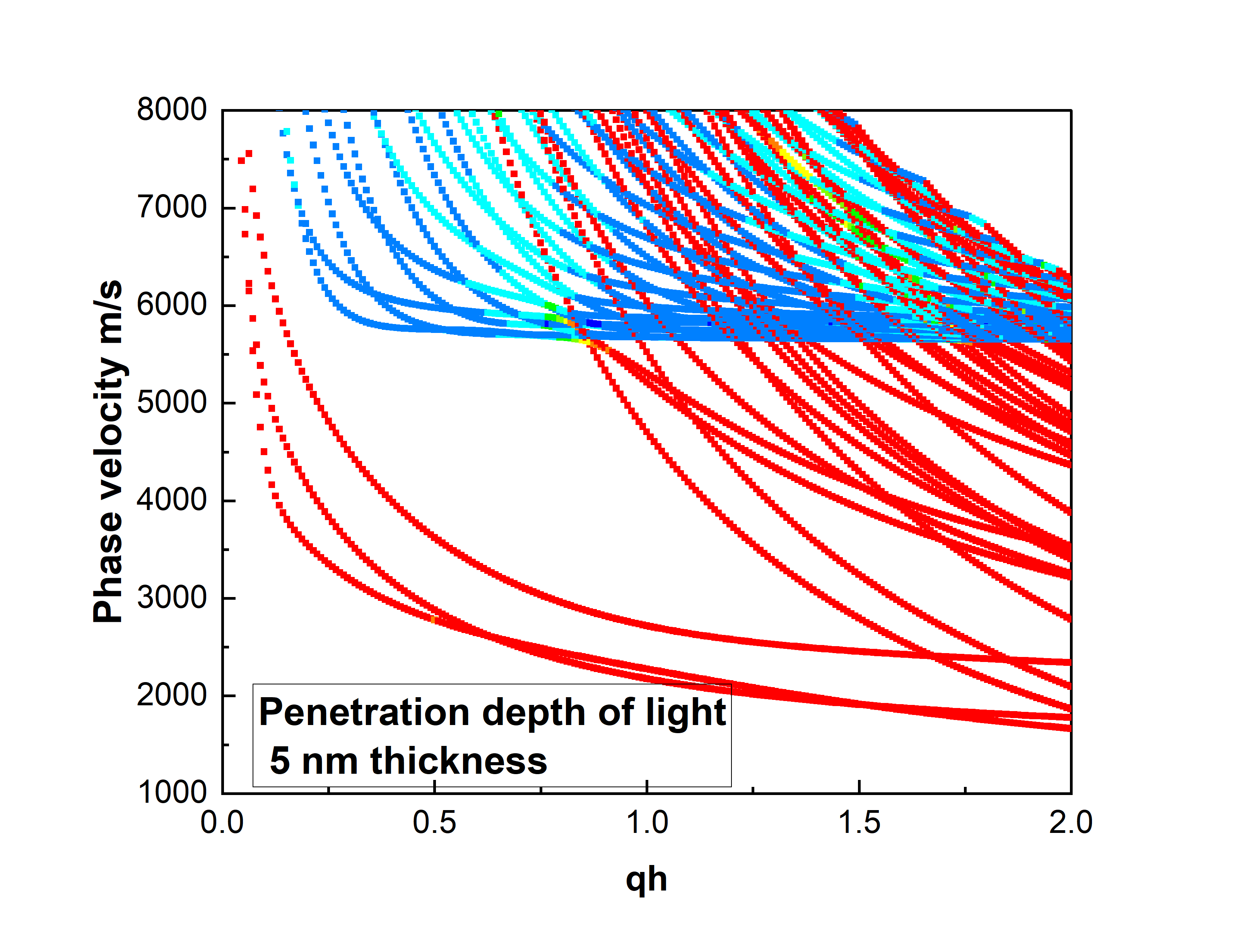
**

ic

hc

gc


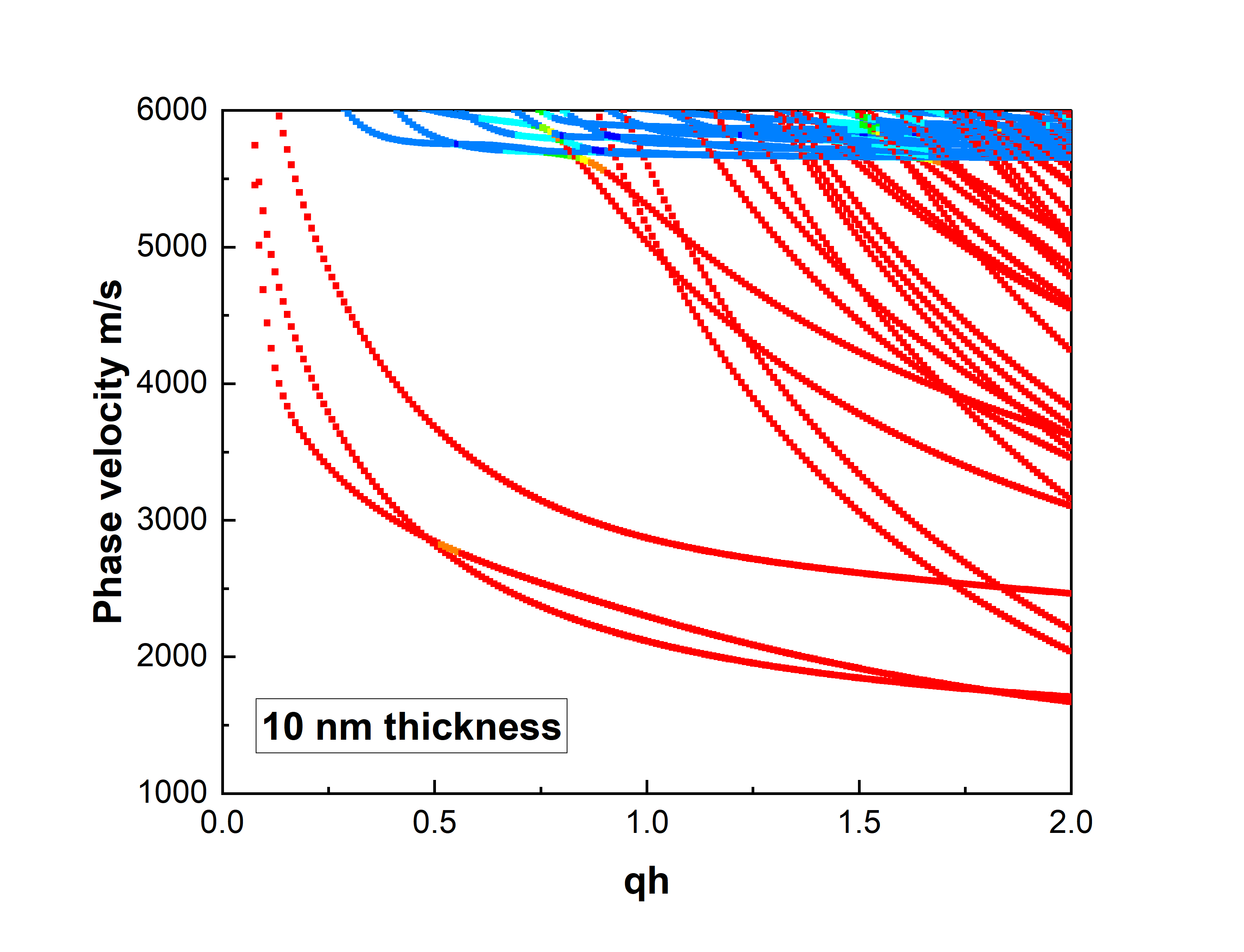

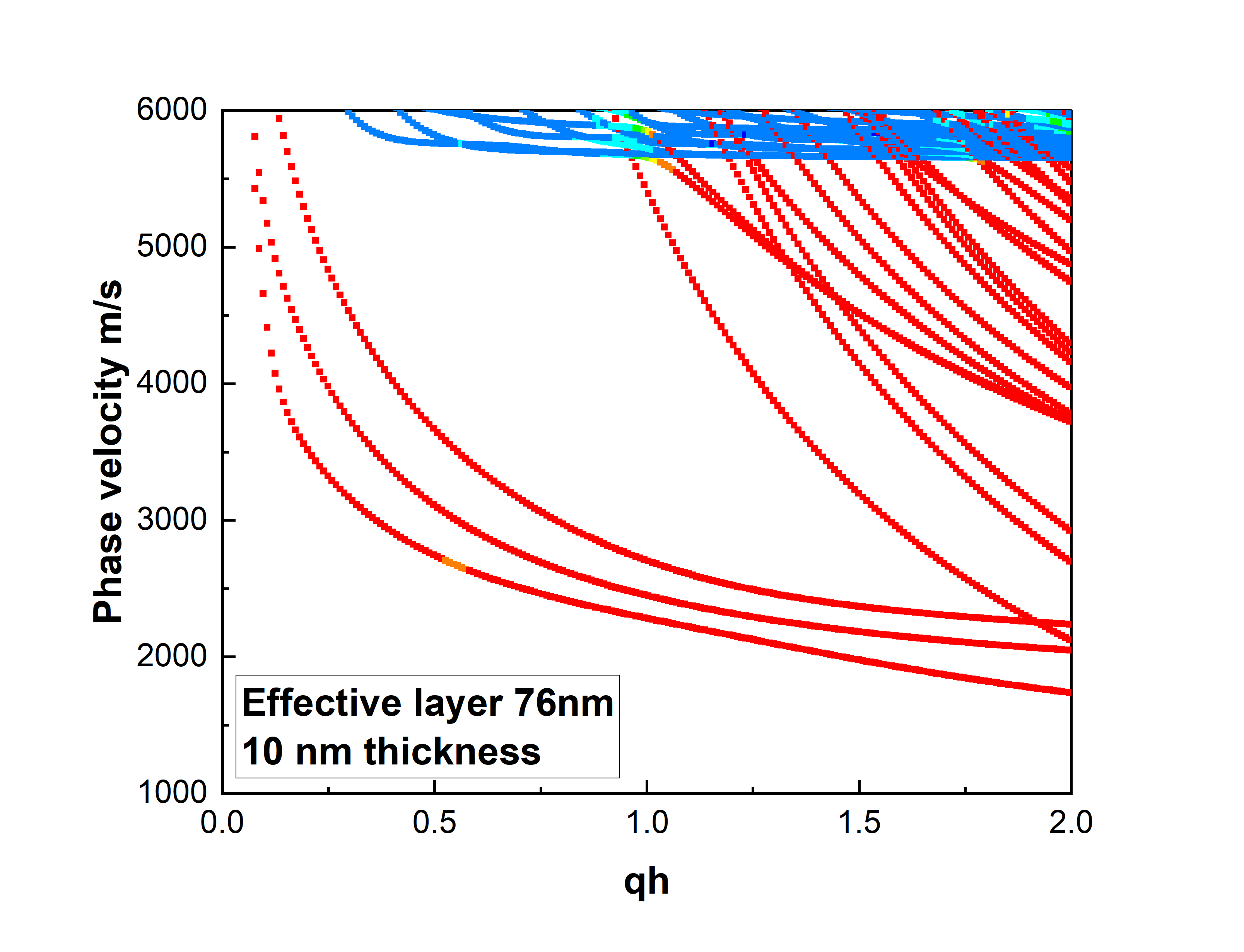
**
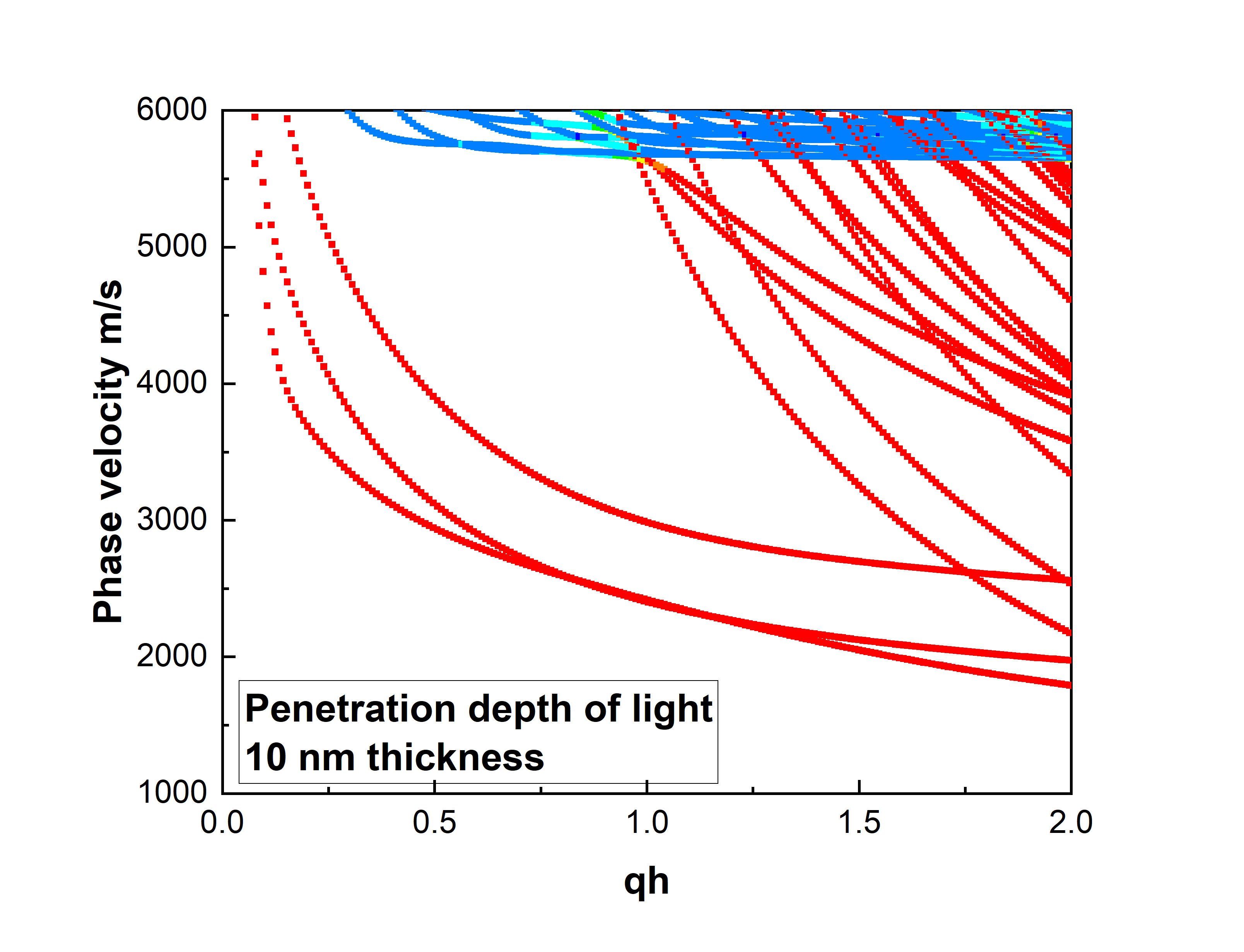
**

lc

kc

j


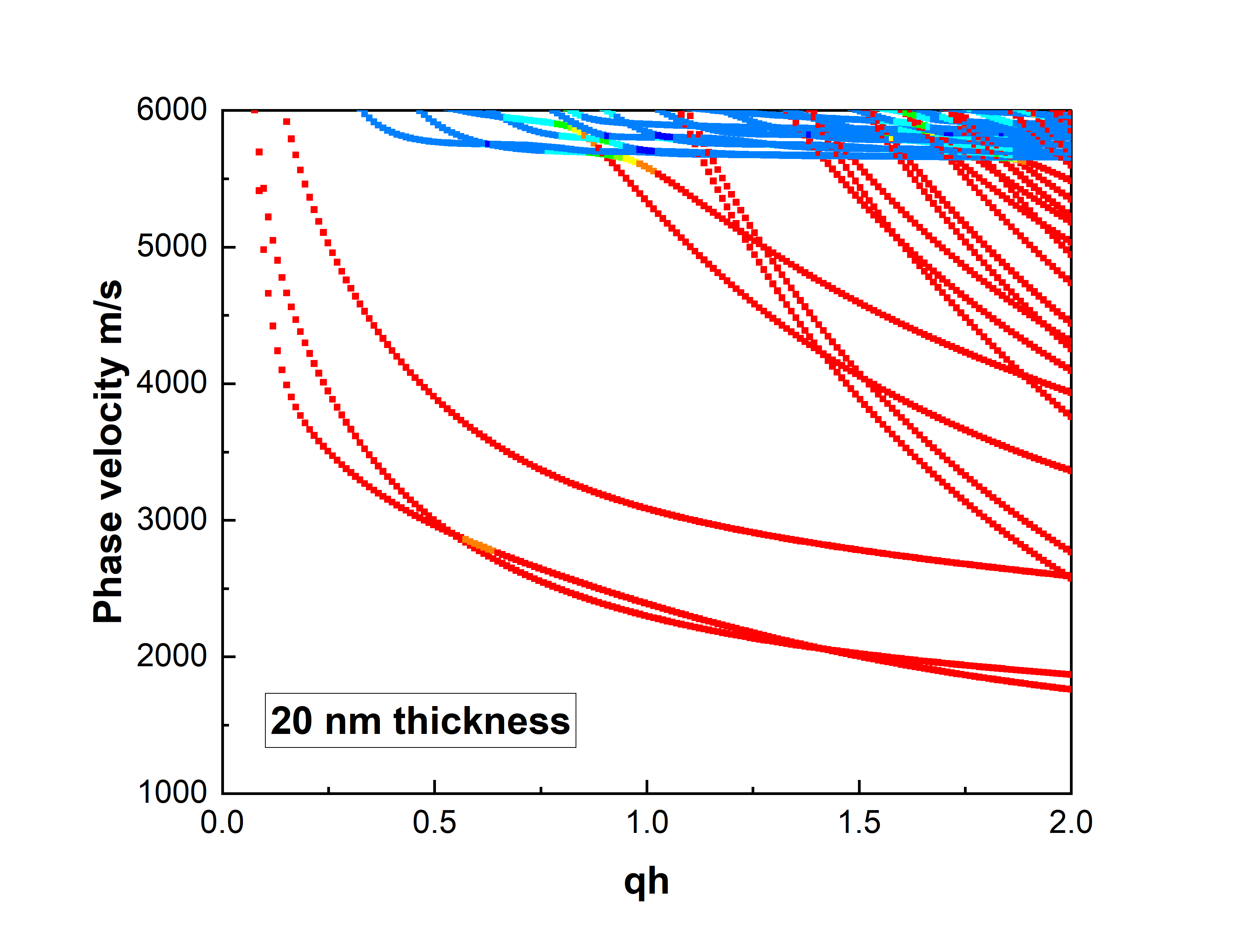

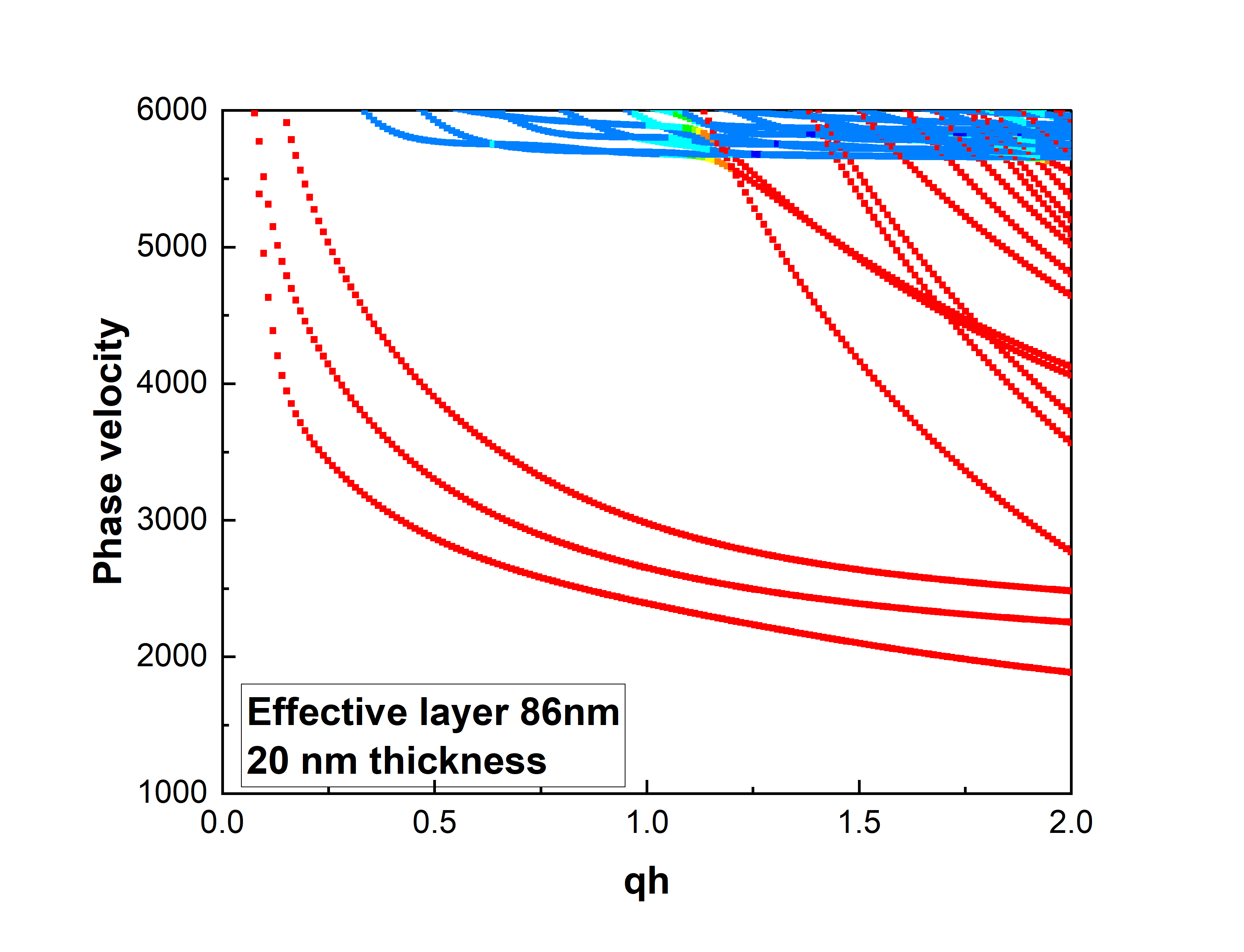
**
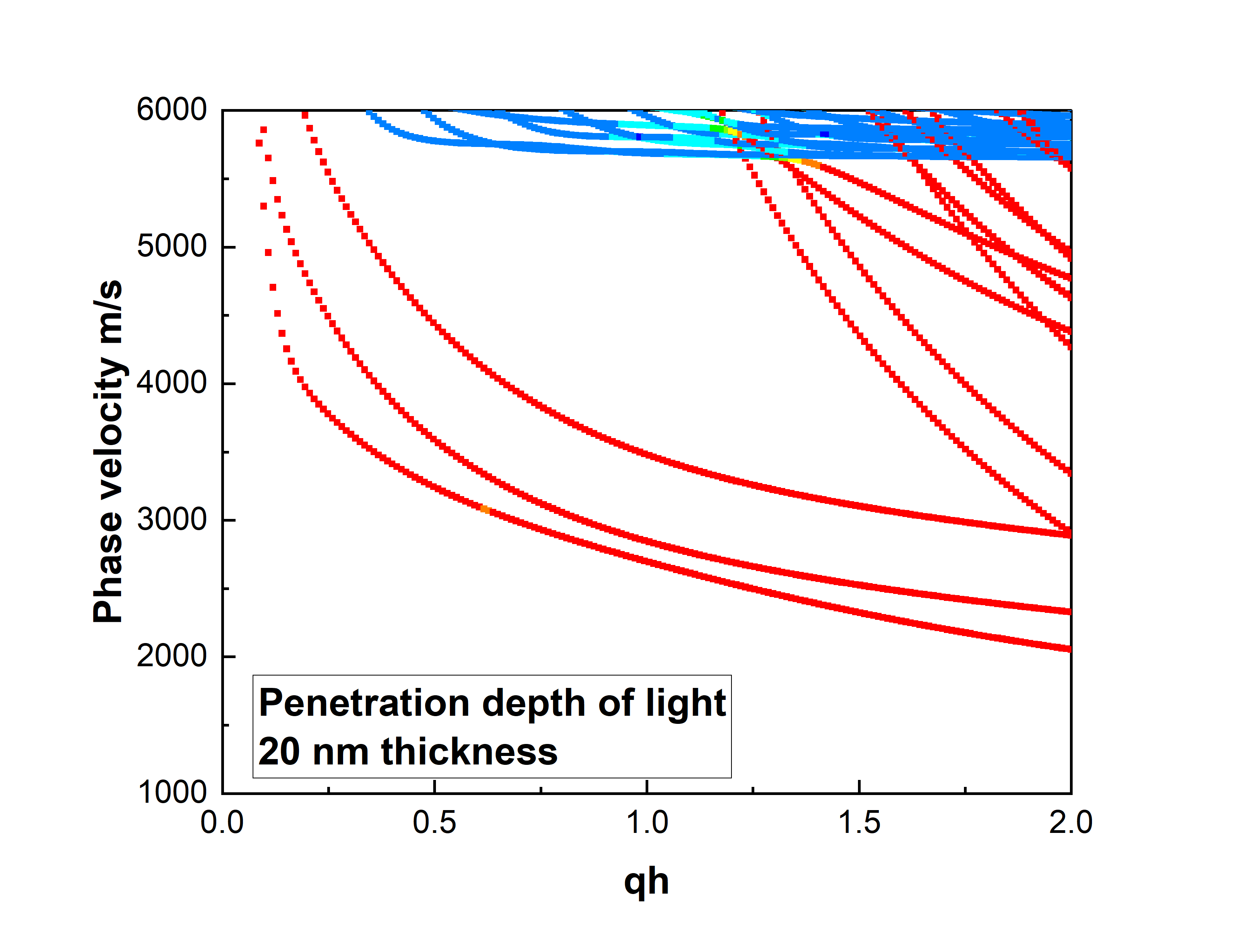
**

Fig. S3. Phase velocity of SAW for samples with different thicknesses of CoFeB.

**S5. Young’s modulus**

Using the elastic tensor values for the sample, we can plot the 2D as well as 3D Young’s modulus (GPa) by using of Elate software [S4].

z

z


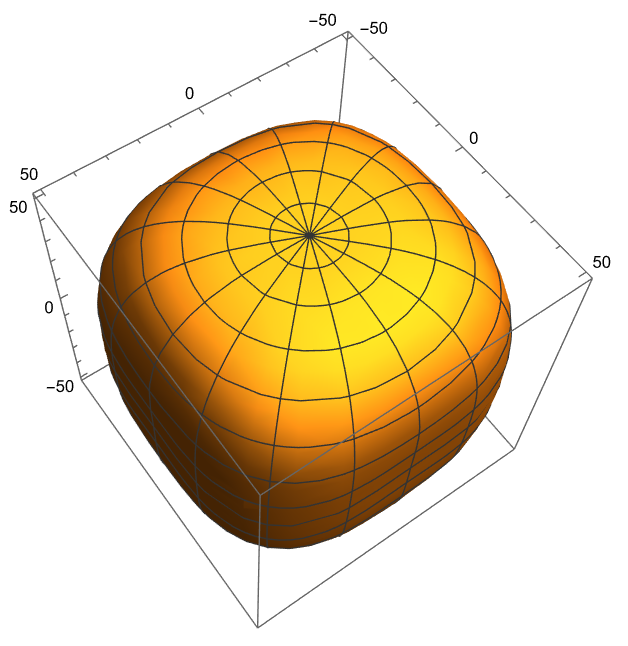

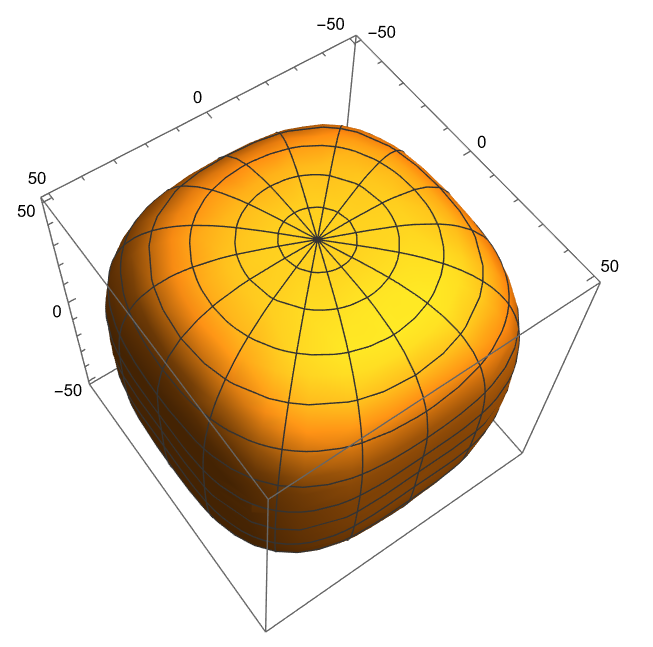


(a)

(b)

x

x

y

y

Fig. S4. Three-dimensional representation of Young’s modulus in GPa for the studied sample with 2 nm of CoFeB. (a) – The multilayer is treated as a single layer with effective elastic parameters; (b) – The light penetration depth is treated as an effective layer.

**S6. Calculation of Zener anisotropy**

The anisotropy of the layers can be calculated using elastic tensors. The equation is shown below[S5,S6]:

$$A= \frac{2c_{44}}{c_{11}-c_{12}}$$

We know that if A = 1, the material is isotropic in nature and if A ≠ 1, the material is anisotropic in nature.

Table S2. The Zener anisotropy values for the samples with CoFeB thicknesses 1.6 nm, 1.8 nm and 1.9 nm, considering both for effective layer and the light penetration depth.

| **CoFeB thickness (nm)** | **Thickness of the effective layer (nm)** | **A** | **Penetration depth of light (nm)** | **A** |
| --- | --- | --- | --- | --- |
| 1.6 | 67.6 | 2.49 | 18.88 | 2.68 |
| 1.8 | 67.8 | 2.48 | 19.05 | 2.58 |
| 1.9 | 67.9 | 2.48 | 19.07 | 2.55 |

**Calculations for the materials which are present in our sample are as below:**

**Silicon**: A = 2×79.9/(165.7 - 63.9) = 159.8/101.8 = 1.57 → anisotropy

**Titanium**: A = 2×43.4/(178 - 80.9) = 86.8/97.1 = 0.89 → less anisotropy

**Gold**: A = 2×42.3/(190 - 161) = 84.6/29 = 2.92 → high anisotropy

**CoFeB**: A = 2×120/(267 - 85) = 240/182 = 1.32 → anisotropy

**S7. Mesh influence**

In all simulations—including those involving multilayer structures, effective medium approximations, and light penetration depth—comparable mesh densities were employed to ensure consistency across datasets. A finer mesh improves the fidelity of numerical results by more accurately capturing geometric details; however, it also increases computational demands. Therefore, a trade-off between accuracy and computational efficiency, particularly in terms of time and memory requirements, must be considered.

Table S3 presents the influence of mesh density on the computation time and the calculated frequency of a surface acoustic wave (SAW) at a fixed wave vector. The data demonstrates that while finer meshes lead to longer computation times, the resulting frequency values remain effectively unchanged, staying within the experimental uncertainty of ±0.04 GHz

Table S3. Comparison of the number of mesh elements and solution time for one wavevector.

| Mesh – number of elements | Solution time (s) | Frequency (GHz) |
| --- | --- | --- |
| 25120 | 139 | 7,338572 |
| 29741 | 177 | 7,338254 |
| 40162 | 324 | 7,334309 |
| 56483 | 553 | 7,334491 |
| 80199 | 1239 | 7,334936 |
| 110533 | 2554 | 7,333742 |

**References:**

S1. Hess P. Surface Acoustic Waves in Materials Science. *Phys Today*. 2002;55(3):42-47. doi:10.1063/1.1472393

S2. Muanenda Y, Oton CJ, Di Pasquale F. Application of Raman and Brillouin Scattering Phenomena in Distributed Optical Fiber Sensing. *Front Phys*. 2019;7. doi:10.3389/fphy.2019.00155

S3. Buckingham MJ. Sound Propagation. In: *Applied Underwater Acoustics*. Elsevier; 2017:85-184. doi:10.1016/B978-0-12-811240-3.00002-3

S4. Gaillac R, Pullumbi P, Coudert FX. ELATE: an open-source online application for analysis and visualization of elastic tensors. *Journal of Physics: Condensed Matter*. 2016;28(27):275201. doi:10.1088/0953-8984/28/27/275201

S5. Anderson OL, Demarest HH. Elastic constants of the central force model for cubic structures: Polycrystalline aggregates and instabilities. *J Geophys Res*. 1971;76(5):1349-1369. doi:10.1029/JB076i005p01349

S6. Ravindran P, Fast L, Korzhavyi PA, Johansson B, Wills J, Eriksson O. Density functional theory for calculation of elastic properties of orthorhombic crystals: Application to TiSi_2_. *J Appl Phys*. 1998;84(9):4891-4904. doi:10.1063/1.368733
